# The cold case of *state transition 7* (*stt7*) mutants of *Chlamydomonas reinhardtii*, solved by whole‐genome sequencing

**DOI:** 10.1111/nph.71473

**Published:** 2026-07-28

**Authors:** Sandrine Bujaldon, Adrien Burlacot, Frédéric Chaux, Francis‐André Wollman

**Affiliations:** ^1^ Laboratoire de Photobiologie et Physiologie des Plastes et des Micro‐algues UMR7141, CNRS‐Sorbonne Université, Institut de Biologie Physico‐Chimique Paris 75005 France; ^2^ Biosphere Science and Engineering Division, Department of Plant Biology The Carnegie Institution for Science Stanford CA 94305 USA; ^3^ Department of Biology Stanford University Stanford CA 94305 USA; ^4^ Division for Marine and Environmental Research Ruđer Bošković Institute Zagreb 10000 Croatia

**Keywords:** *Chlamydomonas reinhardtii*, state transitions, structural variations, *stt7* mutants, whole‐genome sequencing

## Abstract

- The process of State Transitions (ST) corresponds to an STT7 kinase‐driven redistribution of the transmembrane LHCII antenna proteins between Photosystem II (PSII) and Photosystem I (PSI), which results from changes in their phosphorylation state. For the past two decades, two LHCII‐kinase mutants, *stt7‐1* and *stt7‐9*, have been instrumental in the study of STs in *Chlamydomonas reinhardtii*, the former being a null mutant for the kinase but quasi‐sterile in crosses, while the latter, although fertile, has a leaky phenotype.
- Using long‐read sequencing, this study further characterized the genetic lesions of the *stt7* mutant strains through whole‐genome reconstruction and *de novo* chromosome assembly. In addition, two new *stt7* null mutants were generated, one derived by crosses from the original *stt7‐1* and one obtained by Clustered Regularly Interspaced Short Palindromic Repeats (CRISPR)‐associated protein 9 (Cas9) technology.
- This work provides a comprehensive genomic characterization of the original *stt7‐1* null mutant, revealing extensive chromosomal rearrangements and high levels of aneuploidy, associated with increased cell size and meiotic dysfunction.
- Reassessment of their physiology and genetic backgrounds highlights the need for caution in interpreting genetic information. We thus produced more reliable null mutants for the LHCII‐kinase, amenable to genetic crosses for the study of STs in a variety of genetic backgrounds.

## Graphical abstract

Long‐read whole‐genome sequencing revealed extensive chromosomal rearrangements and aneuploidy in a widely used *Chlamydomonas reinhardtii stt7‐1* mutant. The *STT7* gene (shown in red) was found to be split between two rearranged chromosomes, providing a molecular explanation for the long‐standing failure of this mutant in genetic crosses.

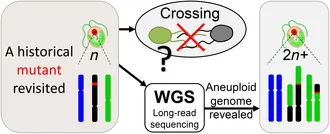

## Introduction

Over the past decades, the green microalga *Chlamydomonas reinhardtii* played a prominent role in our understanding of several major biological processes, such as cilia‐driven motility or oxygenic photosynthesis, due to its amenability to genetic studies (Dutcher, 2023; Grossman & Wollman, 2023). Oxygenic photosynthesis, which converts light energy in electrochemical energy, relies on two photosystems working in series to transfer electrons from water to NADP+. Upon illumination, Photosystem II (PSII) reduces intersystem electron carriers, namely plastoquinones (PQ) to plastoquinols (PQH2), which are oxidized by Photosystem I (PSI). Thus, the redox level of PQ/PQH2 upon illumination depends on the relative rates of light‐induced charge separation by each type of photosystem. Among the regulatory mechanisms that control photosynthesis in a changing environment is the process known as *State Transitions* (ST), ubiquitous among *Viridiplantae* (see Wollman, 2001; Lemeille *et al*., 2010; Minagawa, 2011 for reviews), whereby reversible phosphorylation of antenna proteins, which harvest light excitation energy, migrates from one type of photosystem to the other depending on environmental conditions, thereby modifying their antenna size. The transition between State 1 (ST1) and State 2 (ST2) is triggered by the extent of PQH2 occupancy of the Qo binding site of the cytochrome *b*_6_ *f* complex, an intermediate electron carrier between PSII and PSI (Wollman & Lemaire, 1988). PQH2 binding activates an LHCII‐kinase that phosphorylates several antenna proteins (Vener *et al*., 1995; Zito *et al*., 1999; Dumas *et al*., 2017), which then migrate from PSII to PSI, and back to PSII when dephosphorylated (Bennett, 1977; Allen *et al*., 1981). Therefore, any experimental condition that promotes a full oxidation of the PQ pool in the thylakoid membranes will place Chlamydomonas in ST1, in which the LHCII‐kinase is inactive, leading to the association of dephosphorylated LHCII antenna proteins with PSII. Conversely, experimental conditions leading to a reduction in the PQ pool promote ST2, in which an active LHCII‐kinase phosphorylates a subset of LHCII antenna proteins that associate with PSI. The ST process thus balances the allocation of light excitation energy to each type of photosystem depending on changes in light quality, light intensity or cell demand for ATP, all of which modify the redox state of intersystem electron carriers (Bulte *et al*., 1990; Wollman, 2001). In practice, excitonic coupling of mobile LHCII to either PSII or PSI can be monitored by assessing changes in their apparent antenna size, which leads to a decrease (or ‘quenching’) of the Chl fluorescence emitted by PSII upon a transition from ST1 to ST2. How the reversible phosphorylation of these antenna proteins is achieved by kinase activation is not yet fully understood, but major advances came from pioneering studies in *C. reinhardtii*, which used a genetic screen of random insertion mutants that showed limited or unchanged fluorescence quenching between ST1 and ST2 in several transformants. Among these *st*ate *t*ransition (*stt*) mutants was *stt7‐1*, which displayed a total absence of PSII fluorescence quenching in ST2 conditions, thus being considered a *bona fide* null mutant for STs (Fleischmann *et al*., 1999).

This breakthrough led to the cloning of the *STT7* gene (Cre02.g120250), which encodes a chloroplast‐targeted, membrane‐bound kinase (Depège *et al*., 2003). Since its discovery, two allelic mutants, *stt7‐1* and *stt7‐9*, have been used widely by many laboratories (see, for instance, in recent years (Massoz *et al*., 2015; Dumas *et al*., 2017; Buchert *et al*., 2022; Virtanen & Tyystjärvi, 2023; Riché *et al*., 2024)). These studies also enabled the identification of the orthologous ST‐kinase in plants, called STN7 (Bellafiore *et al*., 2005). Despite their major contribution to studies of ST in Chlamydomonas, the *stt7* mutants suffered from several drawbacks. First, the mutations in the *STT7* locus were reported to be more complex than the expected insertion of the selection marker, but the genetic lesion had remained ill‐defined (Depège *et al*., 2003). Next, the null mutant for the kinase, *stt7‐1*, proved quasi‐sterile in genetic crosses, which prevented its convenient use to combine a null mutation in the *STT7* gene with other mutations of interest when investigating the physiological consequences of a block in ST. Authors thus used the allelic *stt7‐9* mutant, which is amenable to genetic crosses (Cardol *et al*., 2009; Allorent *et al*., 2013). However, this mutant still displayed a weak ST‐associated protein phosphorylation, which was absent in the *stt7‐1* null mutant (Bergner *et al*., 2015). In that respect, it resembled the original *stt7‐2* leaky mutant from which it probably derives (Depège *et al*., 2003). The extent of STs remaining in *stt7‐9* remains a matter of debate but its leakiness questions the relevance of the phenotypes observed in experiments and crosses involving this allele.

Owing to the need for an *stt7* null mutant amenable to genetic crosses to further investigate ST in *C. reinhardtii*, we first aimed at retrieving a suitable mutant from the quasi‐sterile *stt7‐1*. We found mating conditions allowing recovery of some progeny in crosses between *stt7‐1* and a wild‐type (WT) strain. Because of the original report that some genomic rearrangement had occurred in the *stt7‐1* mutant, we implemented a genome‐wide identification of its original genetic lesions that we also assessed in the mutant progeny after one and two backcrosses. We finally used the CRISPR‐Cas9 editing process to generate a new *stt7* null mutant.

## Materials and Methods

### Strains and growth conditions

The strains used in this study are listed in Supporting Information Table S1. WT and mutant strains of *Chlamydomonas reinhardtii* D. A. Dangeard (G.M. Smith) were grown mixotrophically at 25°C in Tris‐acetate‐phosphate (TAP) medium, pH 7.2 (Goodenough, 2023) on a rotary shaker (150 rpm) under continuous light (5–10 μmol photons m^−2^ s^−1^), unless otherwise indicated. CC‐124 was obtained from Chlamydomonas Resource Center (https://www.chlamycollection.org/). The *stt7‐17* CRISPR‐Cas9 mutant deficient in ST has been described in Zaeem *et al*. (2026).

### Gametogenesis and mating

For gametogenesis, cells were grown for 4 d on TAP plates containing one‐tenth the usual amount of nitrogen (0.8 mM instead of 8 mM of NH4). Gametes were then resuspended at *c*. 10^7^ cells ml^−1^ in 2–3 ml sterile water into an Erlenmeyer flask (50 ml) and placed on a rotary shaker (150 rpm) under low light (5–10 μmol photons m^−2^ s^−1^) for 30 min. Plus and minus mating‐type gametes were then mixed in equal volume and left under light (50 μmol photons m^−2^ s^−1^) without agitation. After a minimum of 1 h (depending on the mating efficiency, diploids required more time to mate than WT haploids; Eves & Chiang, 1982), 150–200 μl suspension were spotted on TAP‐3% agar plates which were returned to light overnight. For zygote maturation, TAP‐3% (30 g agar l^−1^) plates were transferred in the dark for a minimum of 7 d. Germination was induced overnight (16–20 h) under very low light exposure (1–2 μmol photons m^−2^ s^−1^), after which the zygotes were dissected using a tetrad dissection microscope (MSM; Singer Instruments, Roadwater, UK).

### State transitions measurements

Before measurements, cells were grown under 5 μmol photons m^−2^ s^−1^ in TAP medium to 2–3 × 10^6^ cells ml^−1^. They were centrifuged at 2000 ***g*** for 5 min at 20°C, and the pellet was resuspended at 5 × 10^6^ cells ml^−1^ in minimum medium (MIN) for *c*. 20 min under growth light intensity.

#### State transition experiments

Cells were placed in oxidizing conditions to achieve ST1, either by incubation for 20 min under vigorous stirring in the dark or by illumination at 50 μmol photons m^−2^ s^−1^ for 20 min in the presence of the PSII inhibitor 3‐(3,4‐dichlorophenyl)‐1, 1‐dimethylurea (DCMU) (10 μM). ST2 was achieved in reducing conditions by placing the cells in anoxia. To this end, glucose (20 mM) and glucose oxidase (2 g l^−1^) were added to cultures in the dark for 20 min, without stirring.

#### Fluorescence induction kinetics

Fluorescence induction kinetics were performed at room temperature using a DeepGreen Fluorometer (JBeamBio, La Rochelle, France) and a SpeedZen ﬂuorescence imaging setup (JBeamBio) (Johnson *et al*., 2009). Maximum efficiency of PSII, *F*_V_/*F*_M_ (*F*_V_ = *F*_M_ – *F*_0_), was determined using dark‐adapted cells. Maximum fluorescence (*F*_M_) was measured upon continuous illumination in the presence of 10 μM DCMU.

#### 77K fluorescence emission spectra

The 77K fluorescence emission spectra were measured with an in‐house built setup. The same cell batch, placed in the presence of 10 μM DCMU in darkness, was used for fluorescence induction at room temperature and 77 K fluorescence studies. For the latter, 10 μl of cells was picked up from the batch, immersed in liquid nitrogen, and the emission spectra were recorded using a Charge‐coupled device (CCD) spectrophotometer (QE6500; Ocean Optics, Orlando, FL, USA), using excitation with a Light‐Emitting Diode (LED) source (LS‐450; Ocean Optics – blue LED, 450 nm).

### Analysis of polypeptides

For protein analysis of thylakoid membrane, 200 ml of cell culture in exponential phase (2–3 × 10^6^ cells ml^−1^) were centrifuged at 2000 ***g*** for 5 min and resuspended in 5 ml of 5 mM HEPES/KOH pH 7.5 containing 10 mM ethylenediaminetetraacetic acid (EDTA) and 0.3 M sucrose and broken in a French pressure cell at 27.56 MPa (4000 lb in^−2^). Thylakoid membranes were recovered using a flotation procedure adapted from (Chua & Bennoun, 1975). In brief, the broken cell suspension was placed on top of a 4 ml 1.8 M sucrose cushion containing 5 mM HEPES/KOH pH 7.5 and 10 mM EDTA, and spun in a swinging bucket rotor at 160 000 ***g*** for 1 h. Thylakoid membranes were collected at the interface between the two layers. They were then resuspended in 10 ml of 5 mM HEPES/KOH pH 7.5 containing 10 mM EDTA and centrifuged at 17 500 ***g*** for 30 min. Protease inhibitors were added in all solutions just before the experiment (Phenylmethylsulfone fluoride; 200 μM, Benzamidine; 1 mM, 6‐aminocaproic acid; 5 mM), and all steps were carried out at 4°C. The thylakoid membranes proteins were solubilized in the presence of 2% SDS and 0.2 M dithiothreitol (DTT) at 100°C for 90 s and then loaded on equal Chl basis (15 μg Chl per lane) and separated by high‐molarity Tris sodium dodecyl sulfate polyacrylamide gel electrophoresis (SDS‐PAGE) with 8 M urea (Piccioni *et al*., 1981). The separated polypeptides were electroblotted onto a nitrocellulose membranes (Amersham Protran 0.1 μm NC) by semi‐dry method. Immunodetection was performed using antibodies against STT7 (Bergner *et al*., 2015) and phosphothreonine (Rintamäki *et al*., 1997). Signals were visualized by enhanced chemiluminescence (ECL – Clarity; Bio‐Rad) and detected with a luminescent image analyzer ChemidocTM (Bio‐Rad).

### Flow cytometry analysis

Cells in exponential growth (2–3 × 10^6^ cells ml^−1^) in TAP medium under continuous light (5 μmol photons m^−2^ s^−1^) were analyzed for cell size and DNA content. A total of 11 000 to 30 000 events per sample were recorded on a MACSQuant Analyzer flow cytometer (Miltenyi Biotec, Bergisch Gladbach, Germany). Cell size was estimated from the side scatter area (SSC‐A) signal (488 nm laser, 488/10 filter), as it provided a better discrimination between cell control batches (haploid vs diploid cells) than the forward scatter area (FSC‐A) under our experimental conditions. The V1‐A channel (405 nm excitation, 450/50 nm detection) was used to evaluate the content of 4′,6‐diamidino‐2‐phenylindole (DAPI)‐stained DNA.

#### DAPI staining

1 ml of cells culture in exponential phase (2–3 × 10^6^ cells ml^−1^) were centrifuged at 2000 ***g*** at RT for 5 min. Pellets were fixed twice in 1 ml of 70% ethanol (70% v/v in MIN) for 15 min, washed, and stained with DAPI (1 mg ml^−1^) to a final concentration of 0.5 ng ml^−1^, followed by a 5 min incubation at RT.

### Genome sequencing and assemblies

#### DNA extraction and size selection

Genomic DNA was extracted and high‐molecular‐weight fragments were preserved as described in Chaux *et al*. (2024). The extraction is followed by a clean‐up using magnetic beads and size selection based on solid‐phase reversible immobilization using the SRE Kit (Circulomics, Baltimore, MD, USA).

#### Library preparation and sequencing

Sequencing libraries were prepared from SRE‐treated high‐molecular‐weight DNA, following Oxford Nanopore Technologies (ONT) protocols for genomic DNA without preamplification. Kits LSK109 were obtained from ONT and Companion Module NebNext from New England Biolabs (NEB, Ipswich, MA, USA). As a minor modification, we started the preparation with 3 μg of DNA. DNA libraries were sequenced on R9.4 Nanopore flow cells in a MinION Mk1B sequencer with default parameters on the MinKNOW operating software, except for the basecalling with Guppy, which was set to real‐time high accuracy. For each run, 500 ng of DNA was loaded and sequenced for 8–12 h. The flow cell was then washed using the Wash kit (ONT) and reloaded with the same sample.

#### Genome assemblies

Reads in FASTQ files were converting in Fasta format (seqkit v.2.1.0; Shen *et al*., 2016) and were mapped onto the reference genome (Phytozome v.6) using minimap2 (v.2.9; Li, 2018). We used Integrative Genome Viewer (IGV; Robinson *et al*., 2011; Thorvaldsdóttir *et al*., 2012) to visualize read mapping. The *stt7‐1 #a6* genome was assembled *de novo* (i.e. reference‐free) using two independent assemblers (Flye and Canu (Koren *et al*., 2017)). Contiguity and synteny were inspected using interactive visualization tools (D‐genies (Cabanettes & Klopp, 2018), Bandage (Wick *et al*., 2015)) in Figs S1, S2, which is an application that performs genome alignment (using Minimap2, a sequence alignment program; Li, 2018) and generates an interactive dotplot. Manual curation was required because assemblers hardly handle aneuploidy. Moreover, some chromosome remained fragmented into few long contigs, due to some large homologous and/or repetitive regions, such as some centromeres and a few transposable elements.

#### *STT7* gene

All reads in FASTA format were subjected to Blast (v.2.12.0) to select those that contained the *STT7* gene sequence (Phytozome v.6) or its fragments. These selected reads were then assembled using Flye (v.2.9). A subsequent BLAST was performed on the obtained contig to align the *STT7* gene sequence, and also the *ARG7* gene (or its CDS in case of the *stt7‐9* strain) or the hygromycin resistance cassette (CRISPR‐Cas9 strain) sequences, to disclose the *STT7* gene in the distinct strains.

Computations were mostly run on the cluster of the French Institute of Bioinformatics (https://www.france‐bioinformatique.fr/en/home/).

### Transformation and complementation of *C. reinhardtii*

The *STT7* gene was amplified by PCR from WT Chlamydomonas genomic DNA. Primers were designed to anneal to regions located 293 base pairs (bp) upstream of the *STT7* start codon and 1598 bp downstream of the stop codon, producing an amplicon of *c*. 8850 bp (all primers used in this study are listed in Table S2). For complementation, mutant cells were grown in TAP medium at 20 μmol photons m^−2^ s^−1^ to early exponential phase (1 × 10^6^ cells ml^−1^), collected by centrifugation at room temperature at 2500 ***g*** for 7 min, and the cell pellet was resuspended in TAP medium containing 40 mM sucrose (TS) to a final cell concentration of 10^8^ cells ml^−1^. For each mutant, the PCR product (*c*. 500 ng) plus 40 μg salmon sperm DNA and 160 ng of the *AphVIII* gene was incubated with mutant cells at 16°C for 20 min. The cells were electroporated at 1 kV, 10 μF capacitance, then incubated at 16°C. After 30 min of incubation, cells were diluted in 5 ml of TS medium. The electroporated cultures were incubated overnight under dim light (5 μmol photons m^−2^ s^−1^) with gentle agitation, then spread on solid TAP medium containing 10 μg ml^−1^ paromomycin and 100 μg ml^−1^ ampicillin and placed under light (30 μmol photons m^−2^ s^−1^). After 2 wk of growth, only transformants harboring the introduced *AphVIII* gene were able to grow. Screening of transformants that have inserted and expressed the *STT7* gene was carried out by fluorescence measurement at room temperature using a Chl fluorescence imaging system (SpeedZen 200; JBeamBio). After placing the transformation plates in anaerobic conditions (by enriching the air with CO_2_ – BD Difco™ *GasPak*™ EZ) for 1 h (conditions favoring ST2) and in the dark (15 min before the measurement), light was then applied to the cells (a condition that activates photosynthesis, leading to a transition to ST1). The evolution of *F*_M_ was then monitored and recorded every minute for 10 min.

## Results

### Phenotypic characteristics of state transitions in two *stt7* allelic mutants from *C. reinhardtii*

**Fig. 1:**
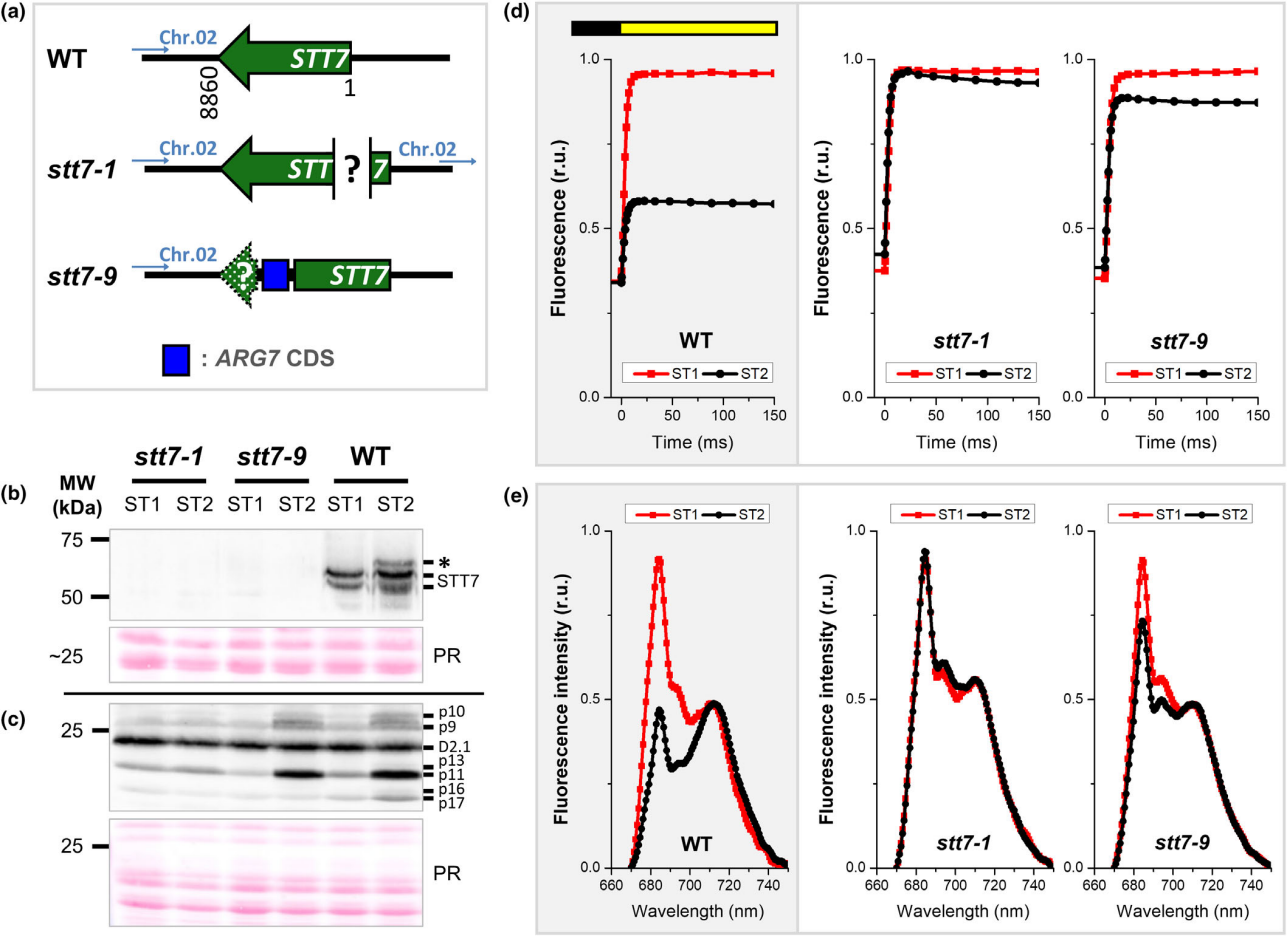
Characteristics of the two original *stt7* allelic mutants of *Chlamydomonas reinhardtii* (*stt7‐1* and *stt7‐9*) compared with a wild‐type strain (WT‐T222). (a) Genomic *STT7* gene (Cre02.g120250; 8.86 kb) representations in the *stt7* mutants; unresolved lesions are indicated by a question mark. (b) Immunoblot analysis of STT7 and (c) phosphorylated polypeptides: thylakoid membranes were isolated in State 1 or State 2 and the proteins were separated on an 8 M urea sodium dodecyl sulfate polyacrylamide gel electrophoresis (SDS‐PAGE) gel (12% ‐b‐ and 12–18% ‐c‐), transferred to nitrocellulose membrane and probed with antibodies against STT7 (b) or phosphothreonine (c). Ponceau Red staining (PR) is provided as a loading control. In (b), the LHCII region (*c*. 25 kDa) is displayed since the 50–75 kDa shows very limited staining. State transitions measurements by (d) emission fluorescence at room temperature in the presence of 3‐(3,4‐dichlorophenyl)‐1, 1‐dimethylurea (DCMU), and (e) low‐temperature fluorescence emission spectra (normalized to the Photosystem I (PSI) emission peak). On blot (b): asterisk (*) corresponds to STT7 phosphorylated form; on blot (c): polypeptide bands p11 and p13 correspond to LHCII type I (LHCBM3/4/6/8/9); p17 to LHCII type III (LHCBM2/7); p16 to LHCII type IV (LHCBM1); p9 to LHCB4 (CP29); p10 to LHCB5 (CP26); D2.1 to D2 (psbD) phosphorylated form.

**Fig. 2:**
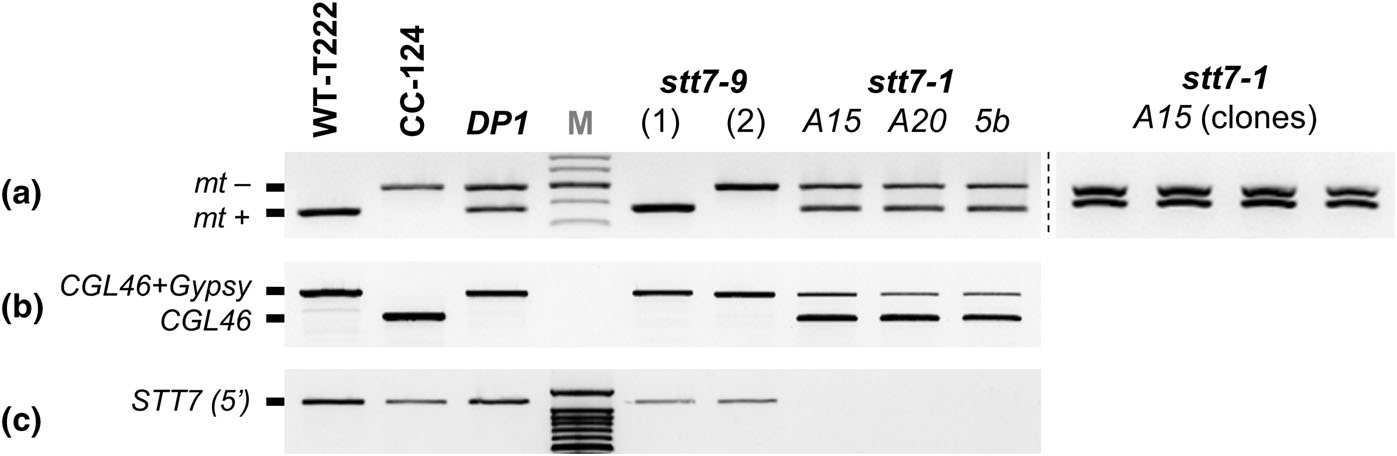
PCR tests of different *stt7* strains/lines (and clones) and control strains in *Chlamydomonas reinhardtii*. Agarose gel electrophoresis of DNA portions amplified by PCR with several sets of specific oligonucleotides (see Supporting Information Table S2): (a) for minus dominance (*MID*) gene (corresponding to *mt* minus) and *FUS1* (*mt* plus), (b) *CGL46* gene and one end of the sequence of *Gypsy‐7a* transposon, and (c) the 5′ region of the *STT7* gene (between Exons 1 and 4). These PCRs were performed on *stt7* mutants, two wild‐type (WT) strains of opposite sexual type, and one diploid strain (*DP1*, *mt+*/*mt‐*). Four sub‐clones of the *stt7‐1*, *A15* strain were also tested for their *mt* (to the right). M, molecular size marker.

The *STT7* gene (Cre02.g120250) encoding the LHC‐kinase in *C. reinhardtii* is located on the minus strand of Chromosome (Chr.) 2. The *stt7* genetic lesions that were previously reported in (Depège *et al*., 2003; Bergner *et al*., 2015) are diagrammatically shown in Fig. 1a. In backcrosses of the *stt7‐9* mutant with a WT strain, the phenotype of a decrease in ST was genetically linked to arginine prototrophy: the *ARG7* gene encoding the argininosuccinate lyase, which was used as a selectable marker gene for random mutagenesis, was found in a gene model that was thus named *STT7*. More precisely, in‐frame insertion of a domain of the *ARG7* cassette resulted in the putative production of a fusion protein between the STT7 kinase (possibly truncated at its C‐terminal end) and the ARG7 protein (Bergner *et al*., 2015). The *stt7–1* mutation also resulted from an insertional mutagenesis using the *ARG7* gene, but the genetic lesion had not been characterized at the molecular level. The *stt7‐1* allele was reported not to be genetically linked to *ARG7* (Fleischmann *et al*., 1999), which confers arginine prototrophy. This observation suggested that the original *stt7‐1* transformant could be used in crosses. In Chlamydomonas, it is a safe practice to backcross mutants to WT to eliminate other possible mutations and use some of the progeny for further investigations. In the case of *stt7‐1*, it seems that the original *stt7‐1* mutant could be crossed but was lost, while the current *stt7‐1* clone comes from a backcross progeny (to be described later). In any case, in the original publications, *stt7‐1* displayed features of some structural rearrangement (Fleischmann *et al*., 1999; Depège *et al*., 2003). In our strains, PCR amplification of the Exon 1 to Exon 4 fragment of the *STT7* gene confirmed its presence in *stt7–9* but not in *stt7‐1*, suggesting the loss of *STT7* in the latter mutant or a possible rearrangement between these two exons (Fig. 2c).

On Fig. 1(b–e), we gathered the main phenotypic characteristics of these two allelic mutants. As shown in Fig. 1(b), the STT7 kinase can be resolved in the WT (WT‐T222) as three electrophoretic bands, which reflect various autophosphorylation states in the 60 kDa region of the immunoblot (Riché *et al*., 2024). These bands are absent in the two LHCII‐kinase mutants, as expected from previous reports (Bergner *et al*., 2015; Cariti *et al*., 2020). Under different electrophoretic conditions, the predicted ARG7‐STT7 fusion product was also detected in the *stt7‐9* mutant, as previously reported (Bergner *et al*., 2015; Fig. S3). We then used a strategy based on distinct chemicals and light pretreatments previously described (see the Materials and Methods section; Wollman & Delepelaire, 1984) to place *C. reinhardtii* cells into either ST1 or ST2. We then assessed the phosphorylation state of the various antenna proteins in the three strains. We separated thylakoid membrane proteins by electrophoresis for immunoblotting using an antiserum to phosphothreonine (Fig. 1c). The phosphorylation level of subunit D2 of the reaction center of PSII, previously identified as D2.1 (Delepelaire & Wollman, 1985), showed no significant changes between the six samples on Fig. 1c. By contrast, several antenna proteins showed a significant increase in their phosphorylation state when WT cells were placed in ST2 as compared to ST1. These included p11 (LHCII type I) and p17 (LHCII type III), as well as p9 (CP29) and p10 (CP26) already described (Wollman & Lemaire, 1988; Bassi & Wollman, 1991). As previously reported (Fleischmann *et al*., 1999), the *stt7‐9* mutant placed in ST2 still displayed a significant increase in p9, p10, and p11 phosphorylation. However, it showed no change in p17 phosphorylation, the reversible phosphorylation of which, together with that of p13, previously has been shown to be instrumental for antenna redistribution between ST1 and ST2 conditions (Delepelaire & Wollman, 1985; Wollman & Lemaire, 1988). Indeed, there is very limited antenna reorganization in this mutant between ST1 and ST2, as viewed by the fluorescence studies described below (Fig. 1d,e), consistent with the key role of p13 and p17 in STs in *C. reinhardtii*.

The phosphorylation pattern of antenna proteins in the *stt7‐1* mutant remained fully unchanged between ST1 and ST2. This pattern was similar to that in WT when placed in ST1, which suggests that the *stt7‐1* mutant is ‘locked’ in ST1 by the absence of LHCII‐kinase activity. When looking at the maximal fluorescence (*F*_M_) levels at room temperature in the three stains (Fig. 1d), here reached upon illumination in the presence of a PSII inhibitor, DCMU, we observed a 60% drop in *F*_M_ in WT cells placed in ST2 (black curve) as compared to ST1 (red curve). This is the expected outcome, since Chl fluorescence at room temperature (RT) is emitted mainly by pigments associated with PSII, which are in larger number in ST1 than in ST2 conditions – where most LHCII antennae proteins have migrated away from PSII to connect with PSI (Delosme *et al*., 1996; Nawrocki *et al*., 2016). By contrast, *stt7–1* cells displayed no significant change at *F*_M_ between the two experimental conditions, while *stt7‐9* underwent a limited drop of *F*_M_ in ST2 as compared to ST1 (20% of the WT; Fig. 1d). Fig. 1(e) shows fluorescence emission spectra at 77K in ST1 and ST2, which resolves a PSII emission peak at 685 nm and 695 nm whereas a PSI emission peak is found at 717 nm (Wollman & Bennoun, 1982). Consistent with the experiments in Fig. 1(d), the WT strain of *C. reinhardtii* showed a marked decrease in the PSII emission relative to that of PSI in ST2 as compared to ST1. Since we normalized the spectra to the PSI emission peak, the huge drop in PSII emission reflects a combination of a decrease in PSII antenna size and an increase in PSI antenna size, as described originally (Wollman & Delepelaire, 1984). No such differences were observed between ST1 and ST2 when using the *stt7‐1* mutant, whereas a very limited drop in PSII emission relative to PSI emission was still observed in the *stt7‐9* mutant. These experiments confirm previous reports that the *stt7‐1* mutant is a null mutant, fully locked in ST1 (Fleischmann *et al*., 1999), whereas the *stt7‐9* is a hypomorphic mutant, severely impaired in STs although it still undergoes significant changes in protein phosphorylation and Chl fluorescence between the two states (Depège *et al*., 2003; Bergner *et al*., 2015; Fig. 1c).

### Mating type, crossing behavior and cell size revealed an aneuploidy in the *stt7‐1*‐derived strains

Since *C. reinhardtii* has a haplobiontic life cycle, laboratory strains are mainly haploid and their mating produces zygotes that represent the only transient diploid stage. Upon zygote germination, siblings return to the haploid state. Since it had been observed that the *stt7‐1* allele is genetically quasi‐sterile (Cardol *et al*., 2009), we made new mating attempts with *stt7‐1* mutants. We started with three *stt7‐1* clones, hereafter referred to as *5b*, *A15* and *A20*, which we had been kept frozen in our laboratory for > 15 yr. Although listed as mating‐type negative (*mt*−) in our records, we first reassessed the mating type of each clone. We used PCR markers, based on gene fragments from the *MT* (Mating Type) locus located on Chr. 06, and the recent determination of the full region (Craig *et al*., 2023). The minus dominance (*MID*) and *FUS1* (fusion) genes are specific to the ‘minus’ and ‘plus’ mating types, respectively (Werner & Mergenhagen, 1998). The results from these PCR analyses of the three *stt7‐1* and two *stt7‐9* clones from our laboratory collection are shown in Fig. 2(a). As control strains, we included two WT strains of opposite mating types, WT‐T222 (*mt+*) and CC‐124 (*mt*−), and a vegetative diploid *DP1* (*mt+*/*mt*−; Drapier *et al*., 2002). The two *stt7‐9* strains exhibited opposite mating types, as shown by PCR and confirmed by agglutination tests with distinct WT strains (Table 1). By contrast, although the three *stt7‐1* clones behaved like *mt‐* in agglutination tests, the PCR analysis surprisingly revealed the presence of the two mating‐type markers *mt*+ and *mt*−, suggesting they were heterozygous for the mating‐type locus. To rule out serendipitous mixing, *stt7‐1* lines were sub‐cloned from single colonies, yet single‐cell clones still amplified both *mt+* and *mt*− markers (Fig. 2a). These observations are not conflicting with the *mt*− behavior of the *stt7‐1* clones in agglutination tests, since the presence of the *MID* gene always results in *mt*− behavior (Ebersold, 1967; Galloway & Goodenough, 1985), as confirmed for the vegetative diploid *DP1* in Table 1A.

**Table 1:** Qualitative mating behavior of *stt7* mutants, wild‐type (WT) and diploid (*DP1*) cells.

Strain	Agglutination		Mating type	Zygote formation^a^	Meiotic product^b^	Cell viability^c^
	With *mt+* WT strain	With *mt*− WT strain				
**A. Control and *stt7* mutant strains**						
WT‐T222	−	+++	Plus	+++	95%	75%
*stt7‐1 5b*	++	−	Minus	+	30%	2%
*stt7‐1 A15*	+	−	Minus	0	/	/
*stt7‐1 A20*	+	−	Minus	0	/	/
*stt7‐9* (*1*)	−	+++	Plus	+++	95%	75%
*stt7‐9* (*2*)	+++	−	Minus	+++	95%	75%
*DP1*	+/−	−	Minus	+	(50%)	(0)
**B. After backcrossing (2)**						
1. *stt7‐1 #1*	++	−	Minus	+++	80%	18%
2. *stt7‐1 #a6*	−	++	Plus	++	80%	15%

In (A), all strains were crossed with WT (*mt+*) and WT (*mt*−) to access agglutination and determine their mating type. Positive agglutination was used to estimate zygote formation, meiotic products, and cell viability after tetrad dissection. In (B), two additional *stt7‐1* strains, obtained after backcrossing, were tested: *#1* from *stt7‐1 5b* × WT+, and *#a6* from *stt7‐1 #1* × WT−.

^a^ Zygote formation: (0) no zygotes; (+) a few zygotes; (+++) regular zygote formation.

^b^ Meiotic product: proportion of zygotes that completed meiosis assessed from a sample of 40 zygotes. For *DP1* × WT cross, only four zygotes were analyzed due to the low fertility of *DP1*. No zygotes were obtained for *stt7‐1 A15* and *stt7‐1 A20* × WT crosses.

^c^ Cell viability: proportion of cells that successfully formed colonies, evaluated from a total of 25 dissected zygotes, corresponding to 100 cells.

We then used allelic variations in *CGL46* (Cre06.g278212), which is located 3.4 Mb away from the *MT* loci on Chr. 06, as another marker to assess aneuploidy. Depending on which WT strains are used in the various laboratories, *CGL46* might house a *Gypsy* transposon (Fig. 2b). Remarkably, the three *stt7‐1* also proved heterozygous for *CGL46* in contrast to *stt7‐9*, which only displayed the *Gypsy* present form. Note that the diploid *DP1* strain showed no heterozygosis at this locus since it was generated in our laboratory (Drapier *et al*., 2002) from *arg‐2* (*arg7‐8*; Eversole, 1956) and *arg‐7* (Gillham, 1965) mutants, which had the same genetic background.

**Fig. 3:**
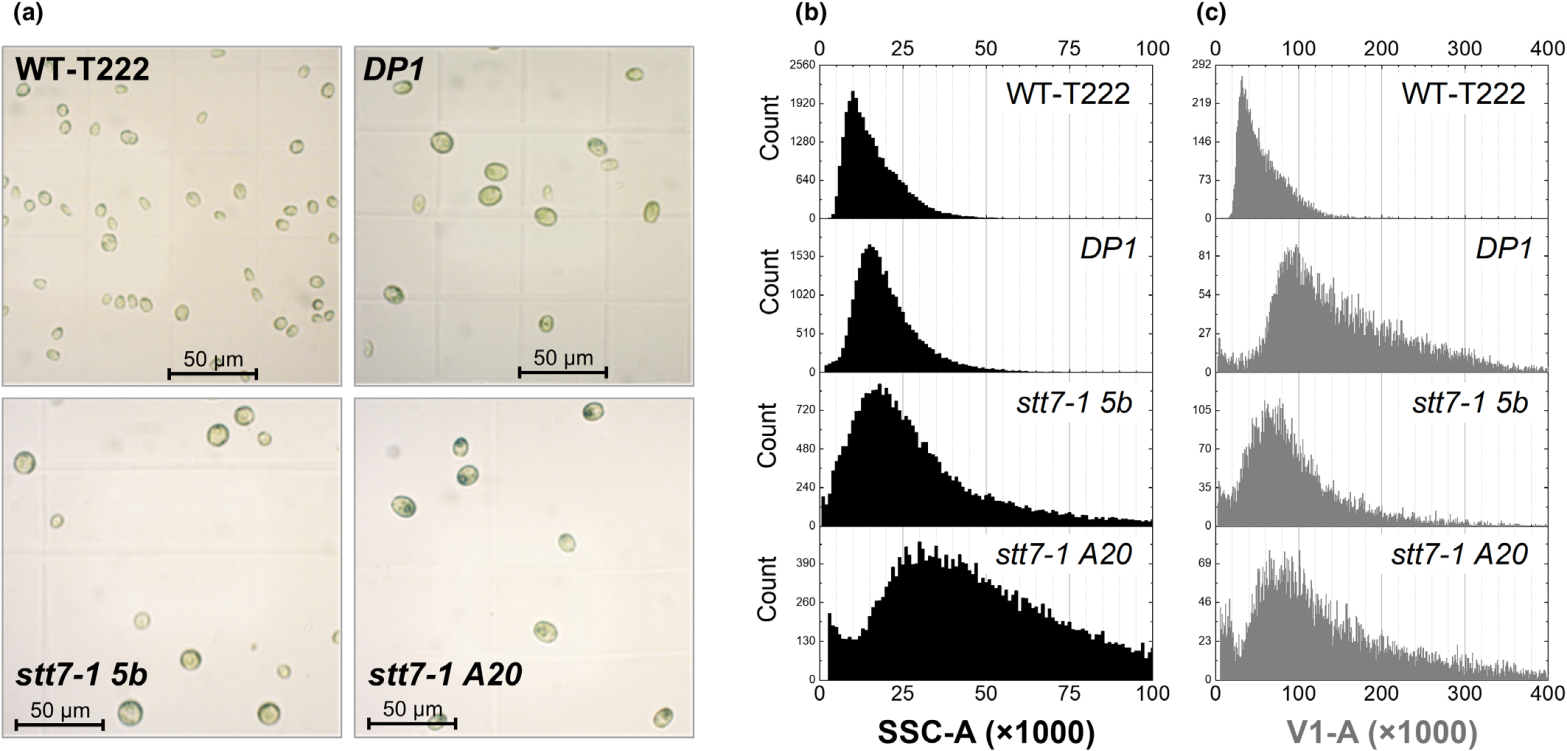
Cellular and nuclear size characteristics of two *stt7‐1* mutants of *Chlamydomonas reinhardtii* compared to a haploid wild‐type (WT‐T222) and a diploid (*DP1*) strain. (a) Optical microscope images of cells in a Malassez counting chamber. (b, c) Flow cytometric analysis (MACSQuant Analyser 10) of asynchronous and exponential phase cultures. Histograms represent: (b) size distribution of 30 000 cells, determined by Side Scatter area (SSC‐A) and (c) fluorescence intensity of 11 000 4′,6‐diamidino‐2‐phenylindole (DAPI)‐stained cells, corresponding to DNA content size distribution, measured in the V1‐A channel (excitation: 405 nm laser; emission filter: 425–475 nm).

Altogether, the results above point to some chromosome duplications in the *stt7‐1* mutant, but the extent of aneuploidy remained unclear. In *Chlamydomonas*, diploid or aneuploid cells tend to have a greater mass and volume than haploid cells, roughly correlating with the nuclear DNA content (Ebersold, 1967). To assess the ploidy differences in these strains, we compared *stt7‐1* clones *5b* and *A20* to the haploid WT strain WT‐T222 and the vegetative diploid *DP1*, using optical microscopy (Fig. 3a) and flow cytometry (Fig. 3b,c). We analyzed 15 000 cells for each strain, both without staining (Fig. 3b) and after adding DAPI, a fluorochrome that binds to double‐stranded DNA (Fig. 3c). Diploid cells (*DP1* strain) were larger (Fig. 3a,b) and contained more DNA (Fig. 3c) than WT cells. Note that the large span of the distribution's right tail represents the different replication stage in the asynchronous population (Seed & Tomkins, 2016). *stt7‐1 5b* clones displayed larger cells than WT‐T222, comparable in size to those from the *DP1* diploid, both of which had a higher DNA content than WT‐T222. Cells from the *stt7‐1 A20* clone were even larger than *DP1* cells (Fig. 3b). Because side scatter (SSC), used here as a proxy for cell size, also reflects intracellular complexity and optical heterogeneity (Moutier *et al*., 2017; Sandmann & Rading, 2024), part of these differences may reflect variations in cellular organization, consistent with the optical microscopy observations of Fig. 3a.

They had a DNA content similar to that in *DP1*, twice as large as in the haploid WT‐T222 cells. As compared to WT, the right tail of the distribution in the other strains was also shifted to higher values and larger span, suggesting that their cell cycle may be altered, for instance by a longer phase of DNA replication. Altogether, the shift of the distribution maxima between the WT and the other strains suggested that aneuploidy in the *stt7‐1* clones may not be restricted to Chr. 06 (Fig. 2a,b) and rather involve other chromosomes. Since aneuploidy generally causes chromosome segregation issues during meiosis (Eves & Chiang, 1982), this might explain the low mating capacity of the *stt7‐1* mutants.

Table 1A summarizes the mating properties of *stt7* mutant strains used in the PCR tests of Fig. 2, in comparison with those of the haploid and diploid control strains, WT‐T222 and *DP1*. The two *stt7‐9* mutant strains were fertile and displayed mating properties similar to those of WT‐T222, whereas *DP1* had low fertility traits, which even were more pronounced in the three *stt7‐1* clones kept in our mutant library. Note that we recovered meiotic products only from crosses with the *stt7‐1 5b* clone, which yielded very little viable progeny. However, the improved mating behavior of this rare progeny of *stt7‐1 5b* allowed us to recover *stt7–1* mutants after one or two backcrosses, which underwent meiosis successfully, releasing a significant proportion of viable progeny (Table 1B; Fig. 8). Whole‐genome long‐read sequencing reveals the structure of the *stt7‐1* mutation in a genome displaying high levels of aneuploidy.

To resolve the mutational landscape of the *stt7* alleles, and more broadly investigate aneuploidy in the entire genome of the *stt7* mutants, we first sequenced two distinct progenies of the original *stt7‐1* mutant (Depège *et al*., 2003), namely *stt7‐1 A20* and *stt7–1 5b* (see the Discussion section for the genealogy of *stt7* mutants), one *stt7‐9* mutant strain, as well as the WT‐T222 strain and the diploid strain *DP1* as controls. Because the *STT7* gene could not be amplified in any of the *stt7‐1* strains (Fig. 2c), we favored long‐read ONT, which allows detection of structural variation (Dutta & Dalal, 2025). Using a BLAST search, we isolated all the reads with a partial or complete match with the region of Chr. 2, which, in the WT, contains the *STT7* gene sequence (8.9 kb), and assembled them using Flye (Kolmogorov *et al*., 2020). Subsequently, a BLAST search was performed on the resulting contigs to map the *STT7* and *ARG7* reference genes, and their flanking regions in the various strains.

**Fig. 4:**
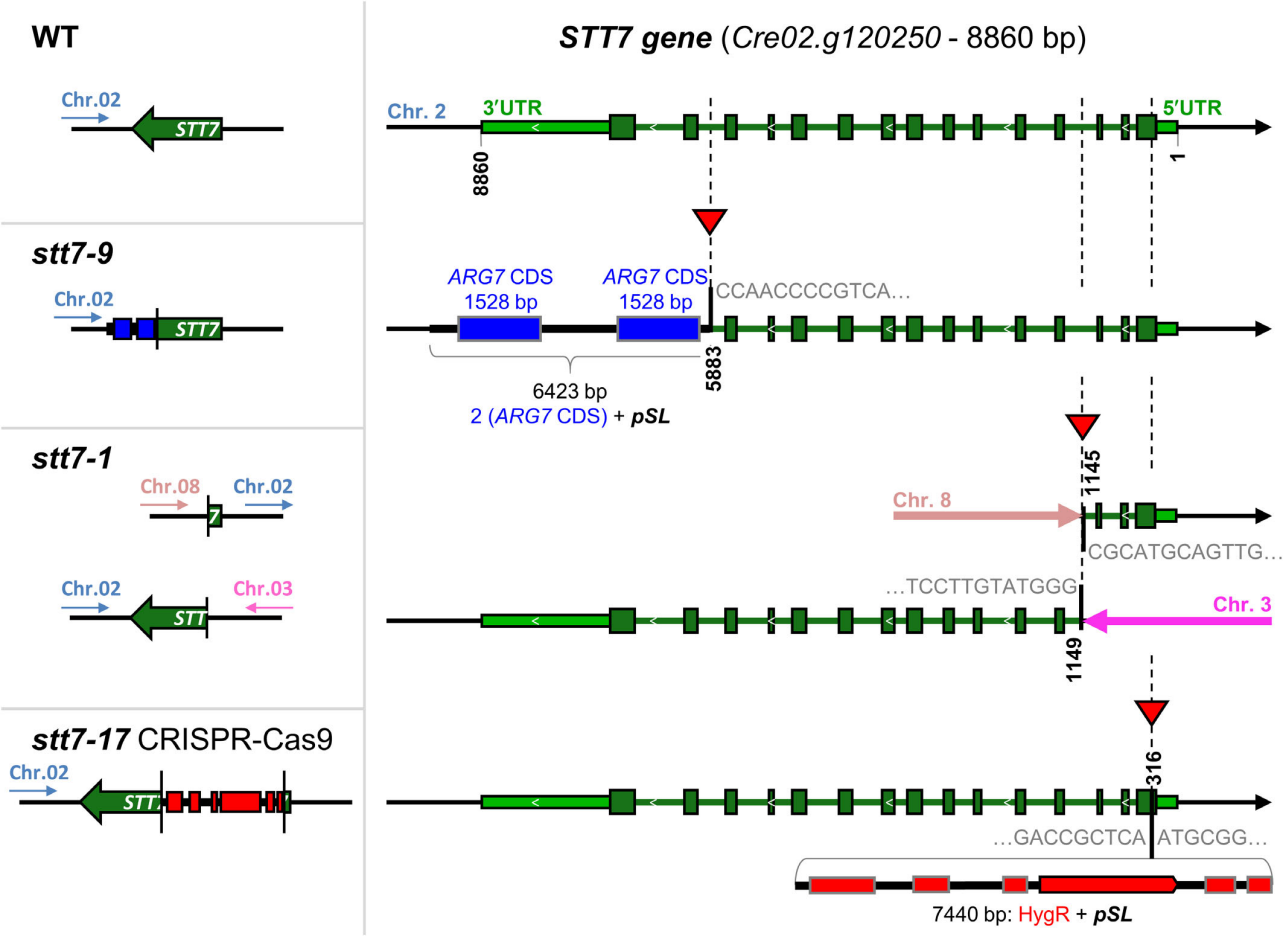
Schematic representation of the *STT7* gene structure in wild‐type (WT) and mutant strains of *Chlamydomonas reinhardtii* used in this study, obtained from Long read sequencing (Oxford Nanopore Technologies (ONT)). On the left, a general representation of the *STT7* gene structure in WT strain and different *stt7* mutant strains. On the right, exons are shown as dark green boxes, introns as green lines, and untranslated regions (UTRs) as light green boxes. In *stt7‐9*, the mutation is located 5883‐bp downstream of the first nucleotide, characterized by a tandem insertion of the *ARG7* coding sequence (CDS, blue boxes) along with the plasmid sequence (*pSL*) used for insertional mutagenesis, and a deletion of the end of the gene; in *stt7‐1*, *STT7* is split into a 1145‐bp 5′ fragment (up to Exon 3) and a 7712‐bp 3′ fragment, which were rearranged into distinct genomic environments, flanked by part of Chromosomes (Chr.) 08 and 03, respectively; in *stt7‐17*, obtained by CRISPR‐Cas9, one hygromycin resistance cassette (*HygR*) and multiple fragments of it (red boxes) are inserted into the first exon at Position 316.

**Fig. 5:**
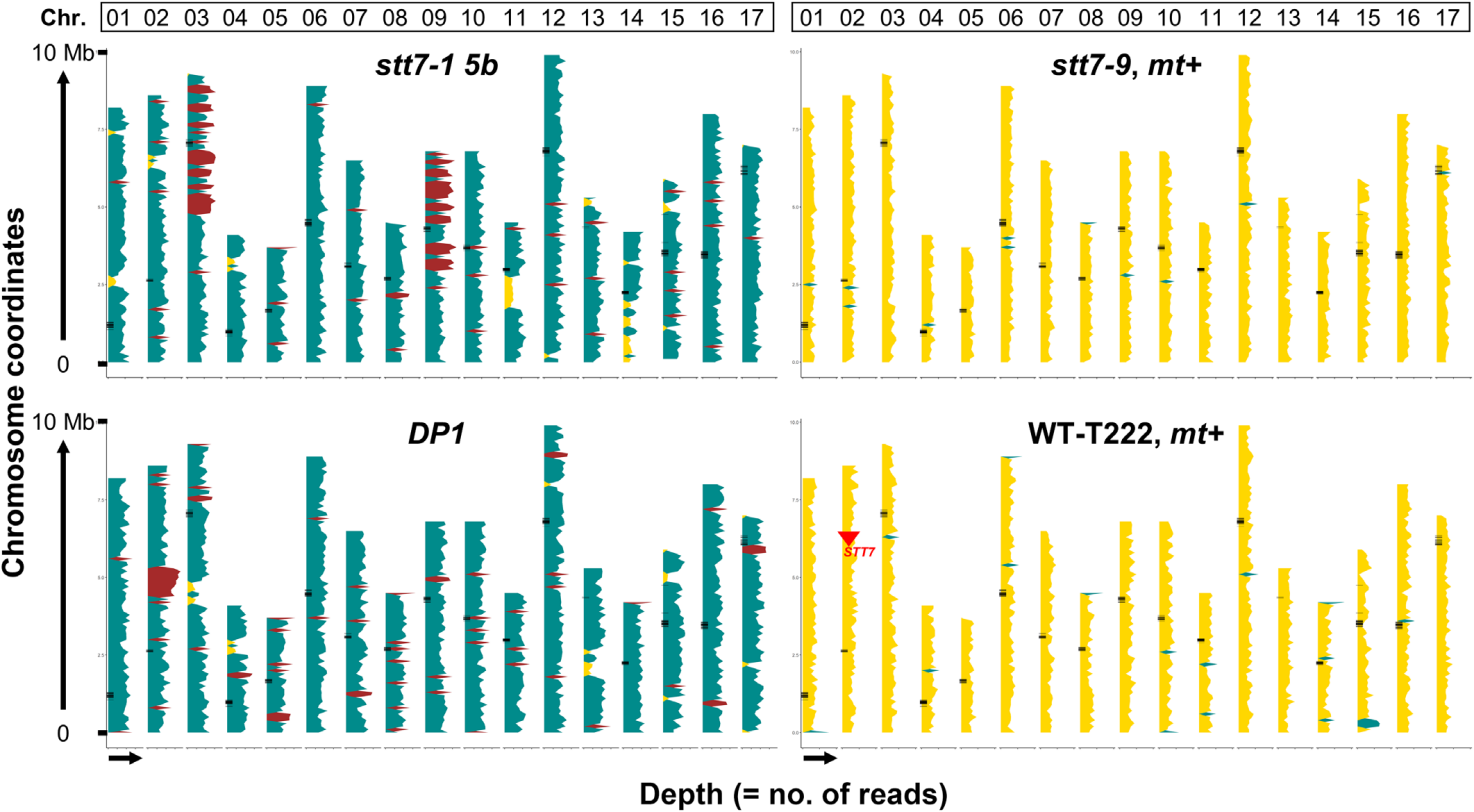
Whole‐genome raw coverage by 1‐kb windows of different strains of *Chlamydomonas reinhardtii*. Graphical representation of sequencing depth (number of reads) along each chromosome. Two *stt7* strains, *stt7‐1 5b* (*mt+*/*mt−*) and *stt7‐9* (*mt+*), are compared with a wild‐type strain (WT‐T222, *mt+*) and a diploid strain (*DP1*, *mt+*/*mt−*). Ploidy levels: haploid (1*n*) in yellow; diploid (2*n*) in dark cyan; triploid (3*n*) in red. Positions of the centromeres are indicated in black to the left of chromosomes (Chr.) 1 to 17. Coordinates of the *STT7* gene (Cre02.g120250): chromosome_02:6189579..6198460 (reverse), marked by a red arrow on the WT‐T222 graph.

**Fig. 6:**
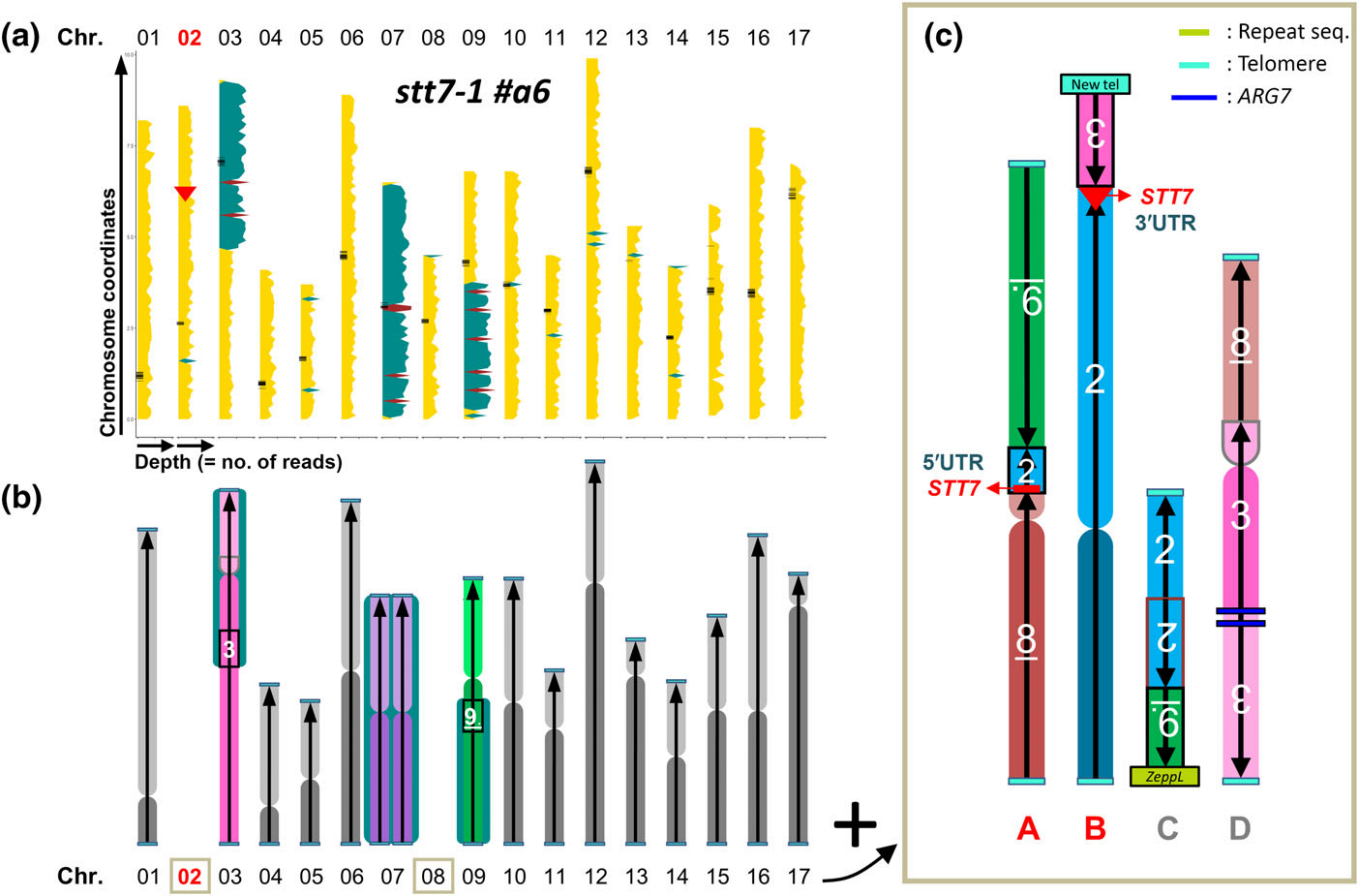
Chromosomal reconstruction of the *stt7‐1 #a6* mutant strain in *Chlamydomonas reinhardtii*. Graphical representations of (a) sequencing depth (number of reads) across each chromosome – where dark cyan represent diploid regions, (b) chromosomes structure; chromosomes are represented with left arms in dark color, extending from the centromere, and right arms in light color (dark cyan boxes indicate diploid regions). Chromosomes (Chr.) 2 and 8 of *stt7‐1 #a6* are not shown in this panel, as they have been fragmented and rearranged with diploid regions from Chr. 3 and 9, forming four novel extra chromosomes, presented in panel (c). The structure and composition of these newly formed chromosomes are described as follows: Chr. A is made of the left arm and the proximal portion of the right arm of Chr. 08 (0–3.0 Mb), a fragment of Chr. 02 (6.1–6.6 Mb) containing the 5′ portion of *STT7* gene (at 6.1 Mb), which connects to the distal part of the left arm of Chr. 09 (0–3.0 Mb, in reverse orientation). Chr. B consists of Chr. 02 left arm and proximal part of right arm (0–6.1 Mb) until the 3′ region of *stt7‐1* allele, which transitions to a short fragment of Chr. 03 right arm (4.1–5.5 Mb, in reverse orientation). A new telomere was identified at the start of this fragment (at 4.1 Mb). Chr. C is composed of the proximal part of Chr. 09 left part (3.0–3.8 Mb) – associated with a repeat sequence region, which is a centromere‐like sequence – and the distal part of Chr. 02 right arm (6.6–8.6 Mb, with inversion of the proximal portion). Chr. D is made of the distal part of Chr. 03 right arm (7.5–9.3 Mb, in reverse orientation) with two insertions of multiple *ARG7* gene and fragments (used for the original insertional transformation), the proximal portion of the left and right arm of Chr. 03 (5.5–7.5 Mb), and the distal part of the left arm of Chr. 08 (3.0–4.5 Mb).

In *stt7‐9*, we found a tandem insertion of the *ARG7* coding sequence (1528 bp) located in *STT7*, 5883‐bp downstream of the *STT7* start codon and a deletion of 2977 bp of the 3′ part of the gene, including the 3′UTR (Fig. 4). Strikingly, in *stt7‐1*, the *STT7* gene was split into two pieces, a short 5′ fragment of 1145 bp (up to Exon 3) and a long 3′ fragment of 7712 bp. The two fragments were further rearranged within a distinct genomic environment, the 5′ and 3′ fragments being flanked by parts of Chr. 8 and 3, respectively (Fig. 4). These observations suggested major chromosome‐scale rearrangements in *stt7‐1* involving at least three chromosomes, which are detailed below and on Figs 5 and 6.

To investigate aneuploidy at the genome‐wide level, we first assessed the sequencing depth in distinct *stt7‐1* mutants, by mapping the reads against the reference genome (CC‐4532, v.6.1). While depth is homogeneous across chromosomes in the WT and in *stt7‐9*, except for highly repetitive regions, such as chromosome ends (Chaux‐Jukic *et al*., 2021), we discovered that, in the two progenies of the original *stt7‐1* mutant (*5b* and *A20*), chromosomes displayed contrasting depth, as also observed – but to a lower extent – in *DP1* (Figs 5, S4). As compared to the main ploidy level (in dark cyan), depth dropped by a factor of two at some chromosomal regions (in yellow) but it increased by a 1.5 factor at some other places (in red). We excluded artefacts due to repetitive, misplaced reads in the two *stt7‐1* strains, because of the standard depth level observed in *stt7‐9* or WT‐T222 (Fig. 5). We thus assumed that the lowest depth level (yellow) corresponds to haploid regions (e.g. left arm of Chr. 14 in *5b*). Therefore *stt7‐1 5b* is mainly diploid (cyan) as is *DP1*, with a few other regions being even triploid (red, for example right arm of Chr. 09 in *5b* and few Mb on Chr. 02 in *DP1*; Fig. 5). A similar analysis with *stt7‐1 A20* also displayed three ploidy levels, diploidy being the main one, although we achieved a more limited sequencing depth (Fig. S4). In good agreement with the diploid characteristics of the *stt7‐1* mutant genome, we found several loci displaying structural differences, including the mating‐type genes (Fig. S5), confirming the co‐existence of two haplotypes in each *stt7‐1* strain (Fig. S6). Whole‐genome sequencing of progenies resulting from one and two backcrosses with WT‐T222 (*stt7‐1 #1* and *stt7–1 #a6*, respectively), showed that they recovered a mainly haploid chromosomal organization for most of their genome (Figs 6, S4). Further characterization of the *#a6* clone showed it mated only with an *mt‐* strain (Table 1B), confirming a homozygous state for *mt +* locus. Detailed analysis of the *stt7‐1 #a6* genome revealed that the remaining diploidy was restricted to Chr. 07 and to the left and right arms of Chr. 09 and 03, respectively (Fig. 6a; note that the latter was a triploid region in the parental strain *stt7‐1 5b*).

To better understand the persisting polyploidy of these chromosomal regions (Fig. 6a), we further characterized the *stt7‐1 #a6* genome by *de novo* assembly (Fig. 6b,c). It displayed the genome sequence of all 17 chromosomes, with two nonrearranged copies of Chr. 07 and rearrangements involving Chr. 02, Chr. 08, and half of Chr. 03 and Chr. 09 (Fig. 6b). Dotplots showed that most chromosomes and chromosome arms from *stt7‐1 #a6* were collinear to those of the WT, but it also provided more detailed information on the genomic rearrangements in *stt7‐1 #a6*, notably those involving the *stt7‐1* mutation site on Chr. 02 (Fig. S1). We could reassemble several chromosome fragments into few long contigs, as four recombined chromosomes based on the presence of putative centromeric and telomeric regions (Fig. 6c):

- Chr. A groups the left arm and the proximal portion of the right arm of Chr. 08, a Chr. 02 fragment starting at the 5′ portion of *STT7* gene, and the distal portion of the left arm of Chr. 09 in reverse orientation.
- Chr. B groups the left arm and proximal part of the right arm of Chr. 02 ending by the 3′ region of the *STT7* gene that is fused with a short fragment of the right arm of Chr. 03 in reverse orientation. Note that a new telomere was identified at the start of this fragment.
- Chr. C groups the proximal part of the left arm of Chr. 09 and the distal part of the right arm of Chr. 02, with an inverted orientation of the proximal portion. A potential new terminal centromere is present (identified by a repeat sequence).
- Chr. D groups the distal part of the right arm of Chr. 03 in reverse orientation, containing two insertions of multiple *ARG7* gene and gene fragments (used for the original insertional transformation (Fleischmann *et al*., 1999)), the proximal segments of both the left and right arms of Chr. 03, and the distal part of the left arm of Chr. 08.

As a consequence of these chromosomal rearrangements, several genes were disrupted, with evidence found in 100% of the reads:

- Cre02.g120250 (*STT7*‐Chloroplast protein kinase required for state transitions) – originally located on Chr. 02; rearranged onto Chr. A and B.
- Cre09.g387023 (describe as a threonine‐specific protein kinase) – originally located on Chr. 02; break point of translocation; rearranged onto Chr. C.
- Cre09.g390100 (S‐adenosyl‐L‐methionine‐dependent methyltransferase) – originally located on Chr. 02; rearranged onto Chr. A and C.
- Cre08.g375050‐*FAP86* (*FAP86*‐Flagellar Associated Protein 86) – originally located on Chr. 08; rearranged onto Chr. A and D.

Regarding the *STT7* gene specifically, which sits on the right arm of Chr. 02 (6.189.579–6.198.460, minus strand) in the WT, we found that it is involved in a two‐way rearrangement in the *stt7‐1* strains (Fig. 6c). Specifically, the gene is interrupted at Position 1145 and fused to Chr. 08, while its downstream part is fused to a fragment of Chr. 03 in opposite orientation. Thus, the *stt7‐1* allele being split between two of these extra chromosomes that cannot match a WT set of chromosomes, any ST null mutant from the *stt7‐1* progeny will retain most of these chromosomal rearrangements.

### Generation of null and fertile *stt7* mutants in Chlamydomonas

We further characterized the *stt7‐1 #a6* clone derived from the original *stt7‐1* mutant for its behavior when placed in either ST1 or ST2. It displayed a null phenotype for ST (Fig. S7). It had no immunodetectable STT7 kinase (Fig. S7a), no differences between ST1 and ST2 in *F*_M_ levels at RT, in fluorescence emission spectra at 77K and in phosphorylation pattern of antenna proteins (Fig. S7b–d). Complementation of *stt7‐1 #a6* with the WT *STT7* allele restored accumulation of the STT7 kinase (Fig. S7a), phosphorylation changes of antenna proteins between ST1 and ST2 (Fig. S7b) and an ST‐dependent fluorescence pattern at RT and 77K, similar to what occurs in the WT‐T222 control strain (Fig. S7c,d). The kinetics of STs in an *STT7‐*complemented strain and an unrescued mutant as a control are shown in Fig. S7(e). Cells were dark‐adapted and maintained under anaerobic (reducing) conditions, which corresponds to ST2 conditions. When these cells were exposed to a transient illumination, which reoxidized the PQH2 pool due to PSI‐mediated electron transfer, the complemented strain displayed an increase in *F*_M_ in the light, indicative of a transition to ST1. When illumination was switched off, there was a decrease in *F*_M_, indicative of transition back to ST2 upon restoration of the anaerobic state. Expectedly, the unrescued *stt7‐1* mutant did not display any of these changes. In the typical experiment shown in Fig. S7(c,d), we note that the complemented strain displayed even larger STs than in the control strain WT‐T222, as viewed by a larger fluorescence quenching in ST2 and a more pronounced increase in antenna protein phosphorylation. This phenotype was consistently expressed in three technical replicates and three biological replicates.

**Fig. 7:**
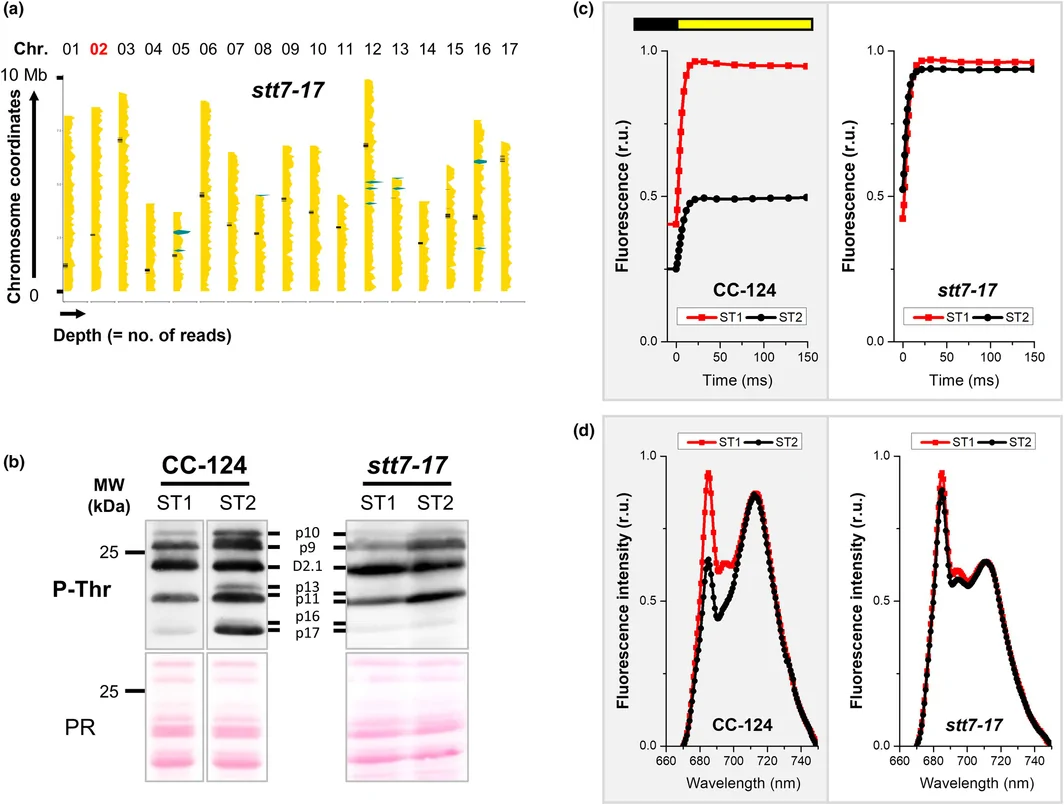
Characteristics of *stt7* CRISPR‐Cas9 transformant in *Chlamydomonas reinhardtii*. (a) Graphical representation of sequencing depth (number of reads) along each chromosome of *stt7‐17* transformant. (b) Immunoblot analysis of phosphorylated polypeptides. Thylakoids membranes were isolated in State 1 or State 2 and the proteins were separated on a 8 M urea sodium dodecyl sulfate polyacrylamide gel electrophoresis (SDS‐PAGE) gel (12–18%), transferred to nitrocellulose membrane and probed with antibodies against phosphothreonine. State transitions measurements by (c) emission fluorescence at room temperature in the presence of 3‐(3,4‐dichlorophenyl)‐1, 1‐dimethylurea (DCMU), and (d) low‐temperature fluorescence emission spectra (normalized to the Photosystem I (PSI) emission peak). The *stt7‐17* clone used in (b–d) was obtained by backcrossing the original *stt7‐17* CRISPR‐Cas9 transformant with CC‐124 wild‐type strain. PS, Ponceau Red staining (PR) is provided as a loading control. p11/p13 correspond to LHCII type I (LHCBM3/4/6/8/9); p17 to LHCII type III (LHCBM2/7); p16 to LHCII type IV (LHCBM1); p9 to LHCB4; p10 to LHCB5; D2.1 to D2 (psbD) phosphorylated form.

Since *stt7‐1 #a6* still showed chromosomal rearrangements and duplications that involve five chromosomes, we also used the CRISPR‐Cas9 technique to generate a novel null mutant in the *STT7* gene. We thus isolated the *stt7‐17* mutant strain generated in the WT‐CC‐125 background, which is also a widely used and well‐characterized WT reference strain (Gallaher *et al*., 2015). As we did above for the other *stt7* mutants, we first examined the structure of the *STT7* gene and its genome ploidy using long‐read sequencing. This analysis revealed an insertion of the hygromycin resistance cassette and fragments of it in the first exon of *STT7* (Fig. 4) and confirmed that its genome is haploid (Fig. 7a). We then checked its ability to undergo genetic crosses with a WT strain of similar genetic background but of opposite mating type – WT‐CC‐124 – and compared its mating behavior before and after a backcross (Table 2). *stt7‐17* exhibited mating characteristics very similar to those of the recipient strain WT‐CC‐125. Additionally, fluorescence measurements under ST1 and ST2 conditions demonstrated that this mutant (a progeny obtained after backcrossing to WT‐CC‐124) was blocked in ST1 when compared to its reference strain (Fig. 7c,d) and there was immunodetectable STT7 kinase (Fig. S3). The ST markers, p13 and p17 (Wollman & Delepelaire, 1984; Fleischmann *et al*., 1999) showed no phosphorylation in either state in the mutant, whereas they are heavily phosphorylated only when WT‐CC‐124 is in ST2 (Fig. 7b; see also Fig. S8). In addition, the phosphorylation pattern of p10 (LHCB5) remained unchanged between the two states in the mutant, whereas it increased in the WT when in ST2.

**Table 2:** Qualitative mating behavior of *stt7‐17* CRISPR‐Cas9 strains.

Strain	Agglutination	Agglutination	Mating type	Zygote formation^a^	Meiotic product^b^	Cell viability^c^
	With CC‐125,*mt+*	With CC‐124, *mt*−				
**A. Control and *stt7* CRISPR‐Cas9 strains**						
CC‐125		+++	Plus	+++	90%	43%
*stt7‐17*	−	+++	Plus	+++	95%	43%
**B. After backcrossing (with CC‐124)**						
*stt7‐17 a3*	−	+++	Plus	+++	97%	58%
*stt7‐17 T2.1*	+++	−	Minus	++	97%	90%

In (A), CC‐125 (*mt+*) was crossed with CC‐124 (*mt*−) to determine mating properties. *stt7‐17* (CRISPR‐Cas9) was crossed with CC‐125 and CC‐124 to assess agglutination, identify its mating type and, based on the positive reaction, estimate zygote formation, meiotic products, and cell viability after tetrad dissection. In (B), two descendant strains from the previous positive cross were tested.

^a^ Zygote formation: (0) no zygotes; (+) a few zygotes; (+++) regular zygote formation.

^b^ Meiotic product: proportion of zygotes that completed meiosis, assessed from a sample of 40 zygotes.

^c^ Cell viability: proportion of cells that successfully formed colonies, evaluated from a total of 25 dissected zygotes, corresponding to 100 cells.

## Discussion

Despite the significance of the identification of the STT7 kinase responsible for STs in microalgal photo‐physiology, the nature of the mutation in the *STT7* gene in *stt7‐1* lines has been overlooked for two decades. The inadequacy of this null mutant for further genetic studies has been bypassed by using leaky counterparts, *stt7‐9* lines, with debatable outcomes. In this work, we leveraged novel sequencing opportunities, extensive genetic efforts, and phenotypic characterizations to re‐open the cold case of *stt7‐1* mutants.

### The *stt7‐1* allele involves a three‐way chromosomal rearrangement

We have established that the lesion of the *STT7* gene in *stt7‐1* mutants is an unexpected rearrangement at the chromosome scale. Specifically, the *STT7* gene is the breakpoint at which Chr. 02 split and fused, on one hand with a portion of Chr. 03, and on the other hand with part of the Chr. 08 (Fig. 6). Chr. 02 and 08 also recombined with Chr. 09 and 03, respectively, which explains why the structure of the genome in *stt7‐1* remained unsolved. We reconstituted these structures based on the newly available technologies of long‐read sequencing. Several clones were individually sequenced and each genome was assembled without using a reference genome (O'Donnell *et al*., 2020; Craig *et al*., 2023) to prevent bias. Because of the high frequency of repeated regions, we did not recover all T2T (‘telomere‐to‐telomere’) chromosomes but manual inspection of the assembly graph and whole‐genome alignments allowed us to recover the genome structure. Chromosome rearrangements as well as heterozygous loci were further validated by retrieving the specific reads and assessing local alignments (BLAST). Similar approaches were recently implemented to investigate genome‐wide recombination in Chlamydomonas, as a result of instability (Chaux *et al*., 2023), but, to our knowledge, this is the first report of such an extensive genome remodeling in Chlamydomonas in the context of resolving the causal mutation of a given phenotype. In this case, since the original transformant was isolated based on the lack of STs, the random breakpoint at the *STT7* locus was likely one of the starting points of the genome shuffling. The mutagenesis method may have triggered other chromosomal rearrangements at the same time in the original cell but this seemingly did not prevent mitosis to form a colony and probably did not induce aneuploidy yet. However, the recombined chromosomes may have compromised meiosis and lead to further alteration upon backcrossing.

### Genealogy of the *stt7‐1* strains: backcrossing led to aneuploid and quasi‐sterile lines

**Fig. 8:**
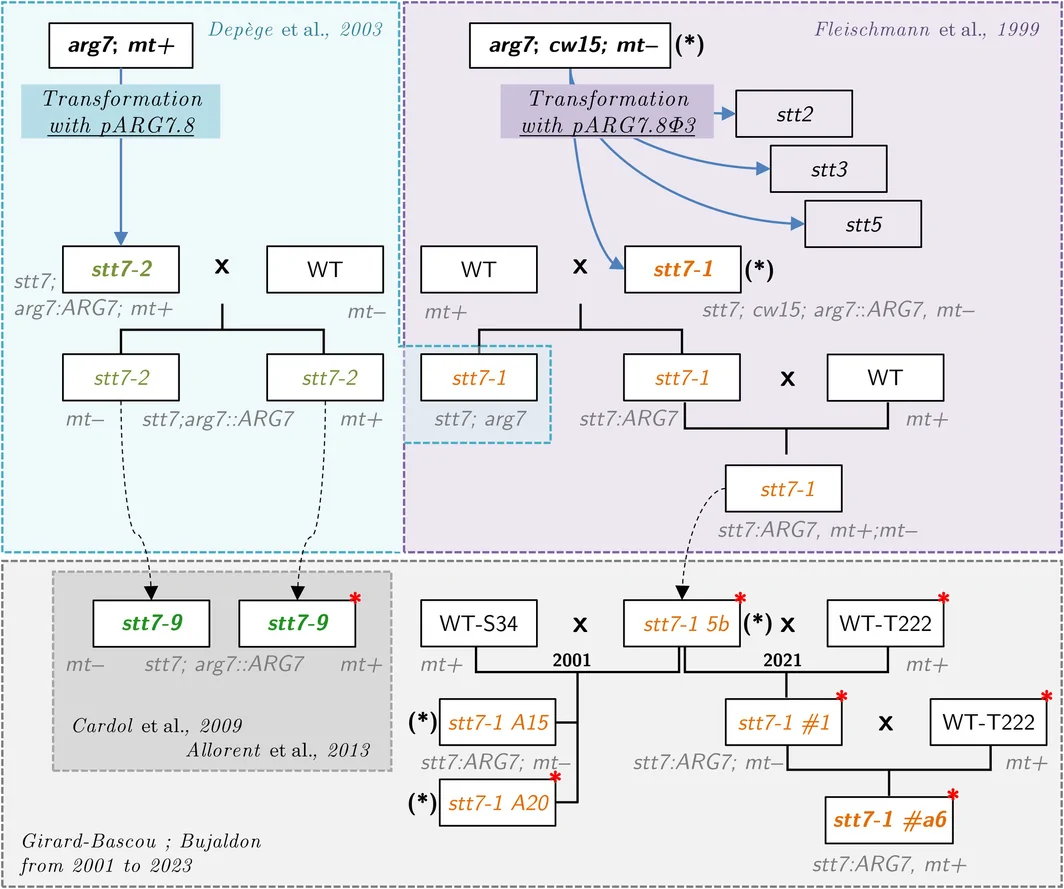
Tentative genealogy of *stt7* mutant strains of *Chlamydomonas reinhardtii* obtained by nuclear transformations and followed by successive backcrosses with selection based on the desired phenotype (defective in state transitions and ability to grow photoautotrophically). Each mutant is labeled with its name and genotype. Two independent mutant strains were initially generated through nuclear transformation: *stt7‐1* (Fleischmann *et al*., 1999) – in purple) was obtained first by transforming the *arg7;cw15* strain (which requires arginine and is cell wall‐deficient) with the *pARG7.8Φ3* plasmid (which restores argininosuccinate lyase (ARG7) activity), while *stt7‐2* (Depège *et al*., 2003) – in blue) was obtained by transforming the *arg7* strain with *pARG7.8* plasmid. These two mutants were backcrossed with a wild‐type strain to ensure a single insertion, determine whether the mutations were tagged and to restore the cell wall (in case of *stt7‐1* mutant). Both mutants displayed the same altered phenotype, no state transitions, which was used as the selection criterion during the backcrossing process. In the case of *stt7‐1*, it was reported to be difficult to backcross and the second backcross revealed that the mutation did not cosegregate with the vector, which contained the *ARG7* resistance cassette (Fleischmann *et al*., 1999). One *stt7‐1;arg7* mutant clone was used to isolate the *STT7* gene by transforming it with a cosmid library containing the *ARG7* gene (Depège *et al*., 2003). In our laboratory (IBPC – in grey), we received these strains from Jean‐David Rochaix's laboratory: *stt7‐1 5b* clone and two clones of «*stt7‐9*» of opposite *mt*, which have been used to perform various crosses. The «*stt7‐2*» and «*stt7‐9*» mutants are identical, each with *ARG7* insertion in the 5′ region as shown by previous studies (Depège *et al*., 2003; Bergner *et al*., 2015) and in this study. The designation «*stt7‐9*» is probably the result of a mislabeling of «*stt7‐2*» during strain transfer. (*): reported as difficult to cross; *: whole‐genome sequencing (Oxford Nanopore Technologies (ONT)).

The genealogy model for the production of *stt7* mutants that we show on Fig. 8, is built on the basis of the *Material and Methods* sections of three seminal papers (Fleischmann *et al*., 1999; Depège *et al*., 2003; Cardol *et al*., 2009) and on our laboratory records. Two independent insertional mutageneses were performed during the period 1998–2002 (Fleischmann *et al*., 1999; Depège *et al*., 2003), both involving the random insertion of an *ARG7* cassette, providing prototrophy for arginine, in an auxotrophic recipient strain *arg7*; the first mutagenesis, which yielded *stt7‐1*, performed in a cell wall‐less mutant background, used the *arg7; cw15* double mutant as a recipient strain, while the second mutagenesis, which yielded *stt7‐2*, used the single mutant *arg7*, which is a cell‐walled strain, as a recipient for transformation. The original *stt7‐1* transformant was subsequently crossed to WT strains to obtain the *stt7* mutation in an arginine prototrophic and a cell‐walled genetic context. During the 2000–2009 time period, this progeny was further backcrossed to the WT strains used in our laboratory, after which the siblings were further disseminated in the Chlamydomonas community. It is remarkable that the *stt7‐9* that we received from the Rochaix laboratory in 2008 and then used extensively in crosses for production of double mutants (Cardol *et al*., 2009; Massoz *et al*., 2015) was not originally described in (Depège *et al*., 2003) but is strikingly similar to the *stt7–2* mutant well described in this publication, which leads us to suggest that the two names refer to the same strain that has been labeled differently at some point.

Much to our surprise, our original *stt7‐1* strains (*5b*, *A15*, *A20*) were mostly diploid and displayed heterozygous loci (Fig. 2), notably in the mating‐type region. This suggests that the original mutant (*mt‐*, recovered around the year 1998) was tentatively backcrossed, in line with early reports (Fleischmann *et al*., 1999; Depège *et al*., 2003). The recombined chromosomes probably did not hamper mating (syngamy) with an *mt +* WT nor did they interfere with chromatid replication at the interphase, but they probably altered meiotic division. Indeed, during metaphase, recombined chromosomes such as the one carrying the split *STT7* gene probably generated detrimental crossing‐overs with WT chromosomes. In many cases, these may have been inappropriate for segregation, thus preventing completion of meiosis, resulting in abortion of zygote maturation. In a few cases, forced resolution of abnormal crossing‐over at anaphase I probably resulted in nonviable daughter cells lacking some chromosomes. However, in other instances, abnormal crossing‐overs may have only prevented segregation during anaphase I without preventing meiosis from proceeding and chromatids from successfully segregating during meiosis II. Such chain of events have been described for the production of allopolyploids, which is relatively frequent since it is assumed to have birthed many hybrid plant species and paleopolyploids (Comai, 2005). In the present case, the zygote would form only two daughter cells followed by mitosis. Thus, the four daughter cells might have been mistaken for a complete tetrad. Backcrossing would not have eliminated the abnormal chromosomes due to their linkage to the phenotype, and autopolyploidy probably reinforced the sterility in the progeny.

### The current *stt7‐1* lines are aneuploid progeny of the original mutants

It was observed that *stt7‐1* proved uneasy to deliver progeny in crosses from its very beginning, as quoted in Depège *et al*. (2003), in agreement with putative genome rearrangements, which should have hampered proper meiosis. Thus, the original mutagenesis probably split the *STT7* gene in two parts, due to genome rearrangements, but it cannot be the source of heterozygosis for the *mt* locus and *CGL46* locus that we detected in *stt7‐1* strains. This aneuploidy was introduced by crossing at subsequent steps of recovery of a cell‐walled *stt7‐1* mutant prototroph for arginine. The translocation events described above may have occurred during the first cross, characterized by reciprocal or nonreciprocal exchanges of chromosomal material between nonhomologous chromosomes during meiosis. Then, after a second cross, the fluorescence screen for an *stt7‐1* phenotype led to the serendipitous selection of a diploid (autopolyploid) vegetative cell, which retained the genetic material from both parents (*mt +* and *mt‐*), thus explaining the difficulty of crossing the *stt7‐1 5b* clone.

### Diploidy is maintained upon mitosis but haploidy is partially restored in the rare meiotic products

The original mutations in *stt7‐1* occurred in haploid receiving strains. However, subsequent crosses involving the original haploid *stt7‐1* yielded production of a vegetative *stt7‐1* diploid as first described by Ebersold in the 1960s. This author observed, as a rare event (< 1% (Ebersold, 1963); < 3–4% (Ebersold, 1967)), that paired gametes fused and divided by mitosis instead of developing into meiosis‐competent zygotes. These strains appeared to be stable diploids, although still undergoing mitotic recombination (Ebersold, 1967).

Abnormal meiosis products were also previously generated in Chlamydomonas, by combining two distinct mutations of a single gene, which can confer prototrophy only when both alleles are maintained by autopolyploidy (Ebersold, 1967; Debuchy *et al*., 1989; Drapier *et al*., 2002). It is remarkable that this constraint on one locus forces the whole genome of such strain (e.g. *DP1*; Fig. 5) to remain fully diploid over countless generations, and not only for the constrained chromosome.

The *stt7‐1* mutants share most of the characteristics of the vegetative diploids originally described by Ebersold: They were heterozygous for the mating type, being able to mate only with haploid *mt+* strain and 80% or more of the meiotic progeny from triploid zygotes from such crosses were nonviable; their persistent diploidy, together with aneuploidy, likely explains the meiotic impairment preventing crosses with other strains. Among the various genes found to be disrupted in the extensively rearranged genome of *stt7‐1* strains, we noted that one version of the Cre09.g397586 gene (located in the diploid region of Chr. 09) is consistently affected in all *stt7‐1* mutants (Fig. S9a): in *5b* and *A20* clones by a deletion at the 3′ end (2176‐bp upstream from the 3′ end), and in *#a6* and *#1* by a 9348‐bp deletion downstream of Exon 7, accompanied by the insertion of a 69‐kb *ZeppL*‐LINE1 repeat sequence (Fig. S9b). These repeat elements constitute the major centromeric sequences in Chlamydomonas, with sizes typically ranging from 51 to 320 kb, with an average of 192 kb (Craig *et al*., 2021, 2023). These observations are consistent with the possibility that disruption of Cre09.g397586 would favor diploidization, maybe by being involved in cell cycle and chromosome segregation. Consistent with this hypothesis, a similar phenomenon was observed in an independent transformation of another recipient strain – which harbored a 175 bp deletion in Exon 8 of this gene – and resulted in diploidization following transformation (K. Wostrikoff, IBPC, personal communication).

Despite this diploidy, we managed to perform several backcrosses (Figs 8, S4). The first viable progeny still displayed diploid traits, whereas more recent backcrosses (since 2020) have yielded offspring clones exhibiting haploid characteristics, particularly at the mating‐type locus, and displaying enhanced mating efficiency. We observed that some supernumerary chromosomes were lost in the progeny (Fig. S4), which probably explains why it has become easier to obtain viable tetrads in subsequent crosses. Nevertheless, the aneuploidy affecting three of the 17 chromosomes persisted and could not be eliminated in the following generation.

### Issues regarding *stt7*‐associated phenotypes

One must be particularly cautious in interpreting a mutant phenotype resulting from a disrupted regulatory process, such as STs. A cascade of functional and biochemical events is attributed to the supramolecular reorganization of the thylakoid membranes upon ST. Some phenotypic traits in *stt7‐1* might stem from the extensive genomic reorganization that has occurred in this strain, in addition to its diploid state, which could have a number of biochemical and functional consequences. As an example of a possible pitfall in the conclusions drawn from a protein kinase‐controlled ST process, due to the extensive gene rearrangements in *stt7‐1*, we identified a gene, Cre09.g387023_4532, located on Chr. 02 of the reference genome (v.6.1) – previous identifier: Cre02.g121350 – encoding a serine/threonine kinase. This gene is disrupted by a translocation breakpoint, splitting it into two fragments that were relocated onto a newly formed chromosome in opposite orientation, very likely resulting in its inactivation. The coding sequence of this gene includes a putative chloroplast targeting signal (predicted by Localizer and WoLF PSORT). In the comparative study by Bergner and coworkers (Bergner *et al*., 2015), the phenotypic differences between the two *stt7‐1* and *stt7‐9* allelic mutants are primarily attributed to a residual STT7 expression in *stt7‐9*, which would explain the very limited – but still significant – changes at *F*_M_ between ST1 and ST2 as well as the low level of phosphoserine or phosphothreonine still detected on substrate proteins for STT7, such as LHCSR3, PETO, TEF23/EGY1 – or even up to a level comparable to that in the WT in the case of LHCB4 – in contrast with their extensively dephosphorylated state in the null mutant *stt7‐1*. However, one cannot exclude a contribution from an alteration of this other serine/threonine protein kinase in *stt7‐1*, which might have independent and/or overlapping activity with that of STT7. Preliminary experiments with two insertion mutants from the Chlamydomonas Library Project (CLiP – (Li *et al*., 2019), see also Table S1) did not exhibit any decrease in STs with respect to that in the WT as viewed by their fluorescence changes between the two states (data not shown). In that respect, we note that, after two backcrosses with WT‐T222, which is accompanied by a significant reduction in polyploidy, the progeny *stt7‐1 #a6* exhibited a slight increase in LHCB4 phosphorylation in ST2 conditions (p9 on Fig. S7b), which also was observed to a higher level in the CRISPR‐Cas9 mutant *stt7‐17* (backcross‐derived clone; Fig. 7b) but not in the original *stt7‐1* mutant (Fig. 1c). The activity of this other kinase encoded by Cre09.g387023 could be responsible for these differences. The haploid *stt7‐1 #a6*, derived from the original *stt7‐1* diploid, should be useful to address these phenotypic concerns. It still shows the same fragmentation of the *STT7* gene and the same Chr. 02 rearrangements but in a haploid context. When used in comparison with its proper control, the complemented strain (*stt7‐1 #a6:STT7*), *stt7‐1 #a6* should be used to assess the reliability of previous studies that were based on the phenotype of the original *stt7‐1* diploid strain.

Whether aiming at a better understanding of the physiological significance of ST or at further investigations of the contribution of the various antenna proteins in this process, investigators will need to place an *stt7* null mutation in a variety of genetic contexts. In this respect, using the *stt7‐1 #a6* mutant in crosses will remain highly challenging due to its rearranged genome and the presence of extra chromosomes. For such issues, the *stt7‐17* null mutant generated by the CRISPR‐Cas9 technology that we described here, with a neat genetic lesion in the *STT7* gene, should prove most useful. It is readily amenable to genetic studies of STs since it can be used smoothly in crosses in *Chlamydomonas reinhardtii*.

## Competing interests

None declared.

## Author contributions

SB performed the investigation, formal analysis, and wrote the original draft of the manuscript. SB, FC and F‐AW contributed to the conceptualization of the study and the development of the methodology. F‐AW supervised the project. SB, AB, FC and F‐AW contributed to the review and editing of the manuscript.

## Disclaimer

The New Phytologist Foundation remains neutral with regard to jurisdictional claims in maps and in any institutional affiliations.

## Supporting information


**Fig. S1** Comparison of genome assemblies – Dotplot analysis of chromosome 2 sequences from CC‐4532 (WT genome reference v6) and *stt7‐1#a6*: Alignment and Variations.
**Fig. S2** Chromosome 9 structure and node visualization with Bandage: Insights from *stt7‐1 #a6*.
**Fig. S3** Immunoblot analysis of STT7 of the three *stt7* allelic mutants used in this study.
**Fig. S4** Long‐read (Nanopore) whole‐genome sequencing depth across each chromosome.
**Fig. S5** Sequencing coverage and read alignments across two regions of the mating‐type locus.
**Fig. S6** Sequencing coverage and read alignments of two chromosomes regions.
**Fig. S7** State Transitions characteristics of *stt7‐1 #a6* clone and its complemented strain.
**Fig. S8** Immunoblot analysis of phosphorylated polypeptides.
**Fig. S9** Cre09.g397586 gene structures in *stt7‐1* and *stt7‐9* strains.
**Table S1** Chlamydomonas strains used in this study.
**Table S2** Primers used for the PCR experiments.
Please note: Wiley is not responsible for the content or functionality of any Supporting Information supplied by the authors. Any queries (other than missing material) should be directed to the *New Phytologist* Central Office.

## Data Availability

Nanopore read FASTQ files generated in this study have been submitted to the European Nucleotide Archive (ENA; https://www.ebi.ac.uk/ena/browser/home) under project accession no.: PRJEB110272. The mutant cell lines are available on request from authors. The *stt7‐17* mutant is available in the Chlamydomonas Resource Center (CC‐6272; www.chlamycollection.org).
