# Supplementary material for: The cold case of state transition 7 (stt7) mutants of Chlamydomonas reinhardtii, solved by whole‐genome sequencing
Source: New Phytol. 2026 Jul 28;252(1):145–62. doi: 10.1111/nph.71473 (PMC13539953; doi:10.1111/nph.71473)
Supplement: Supplementary file 1 [file NPH-252-145-s001.pdf]

New Phytologist Supporting Information

Article title: The cold case of *state transition 7* (*stt7*) mutants of *Chlamydomonas reinhardtii*, solved by whole-genome sequencing.

Authors: Sandrine Bujaldon[1], Adrien Burlacot[2,3], Frédéric Chaux[4,*], Francis-André Wollman[1,*]

Article acceptance date: 10 July 2026

Affiliations:

[1] Laboratoire de Photobiologie et Physiologie des Plastes et des Micro-algues, UMR7141, CNRS-Sorbonne Université, Institut de Biologie Physico-Chimique, Paris, 75005-France

[2] Biosphere Science and Engineering Division, Department of Plant Biology, The Carnegie Institution for Science, Stanford, CA 94305, USA

[3] Department of Biology, Stanford University, Stanford, CA 94305, USA

[4] Division for Marine and Environmental Research, Ruđer Bošković Institute, Zagreb, 10000 Croatia

[*] Corresponding authors

Correspondence: fchauxj@irb.hr, wollman@ibpc.fr

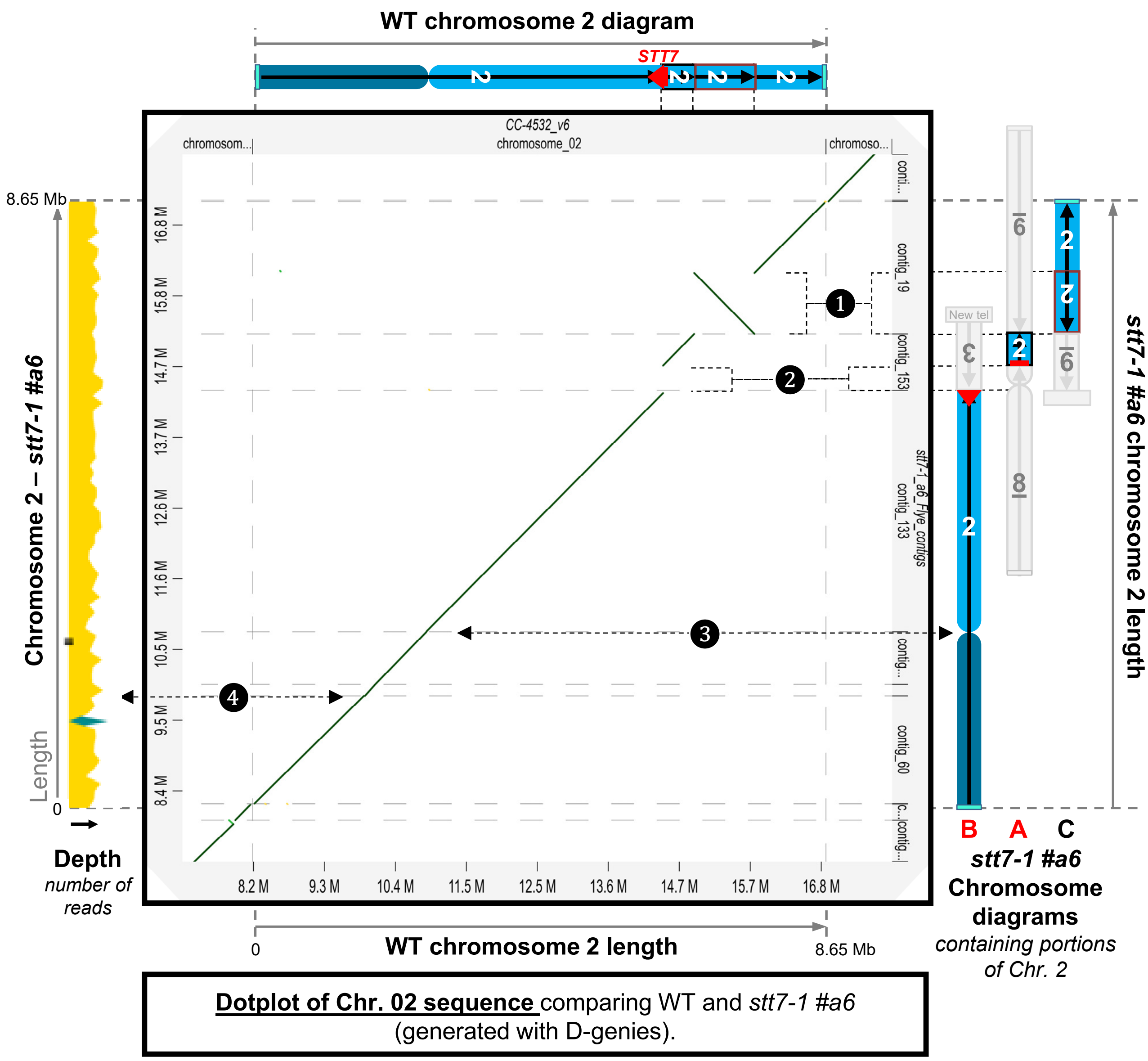

Figure S1. Comparison of genome assemblies - Dotplot analysis of chromosome 2 sequences from CC-4532 (WT genome reference v6) and *stt7-1#a6*: Alignment and Variations.
Dotplot was generated using Minimap2 aligner, to compare the Flye FASTA file of *stt7-1 #a6* (de novo assembly) with the reference genome FASTA file (CC-4532 v6), and visualized using D-GENIES (black box at the center of the figure). Above this plot is a diagram of Chr. 2 from the WT (*STT7* marked with a red arrow), with its length and orientation shown below. The left and right sides relate to *stt7-1 #a6*, displaying the sequencing depth of Chr. 2 and diagrams of all chromosomes containing segments of Chr. 2, providing additional context and data visualization.
The upper and right edges of the plot display the sequence names (from WT CC-4532 and *stt7-1#a6* respectively), while the left and bottom sides show the sequence size scales. Alignment matches are presented as colored lines on the plot (with similarity values above 75%). Several breaks are visible (different contigs), corresponding to either sequence inversions (❶), gaps caused by insertions—reflecting a shift in sequence alignment in the mutant (❷)—transposon insertions (❹), or repeated regions such as centromere (❸). Note that ploidy cannot be visualized in this representation; therefore, another bioinformatics tool, such as Bandage, is required for ploidy analysis (see Supplemental Figure S2).

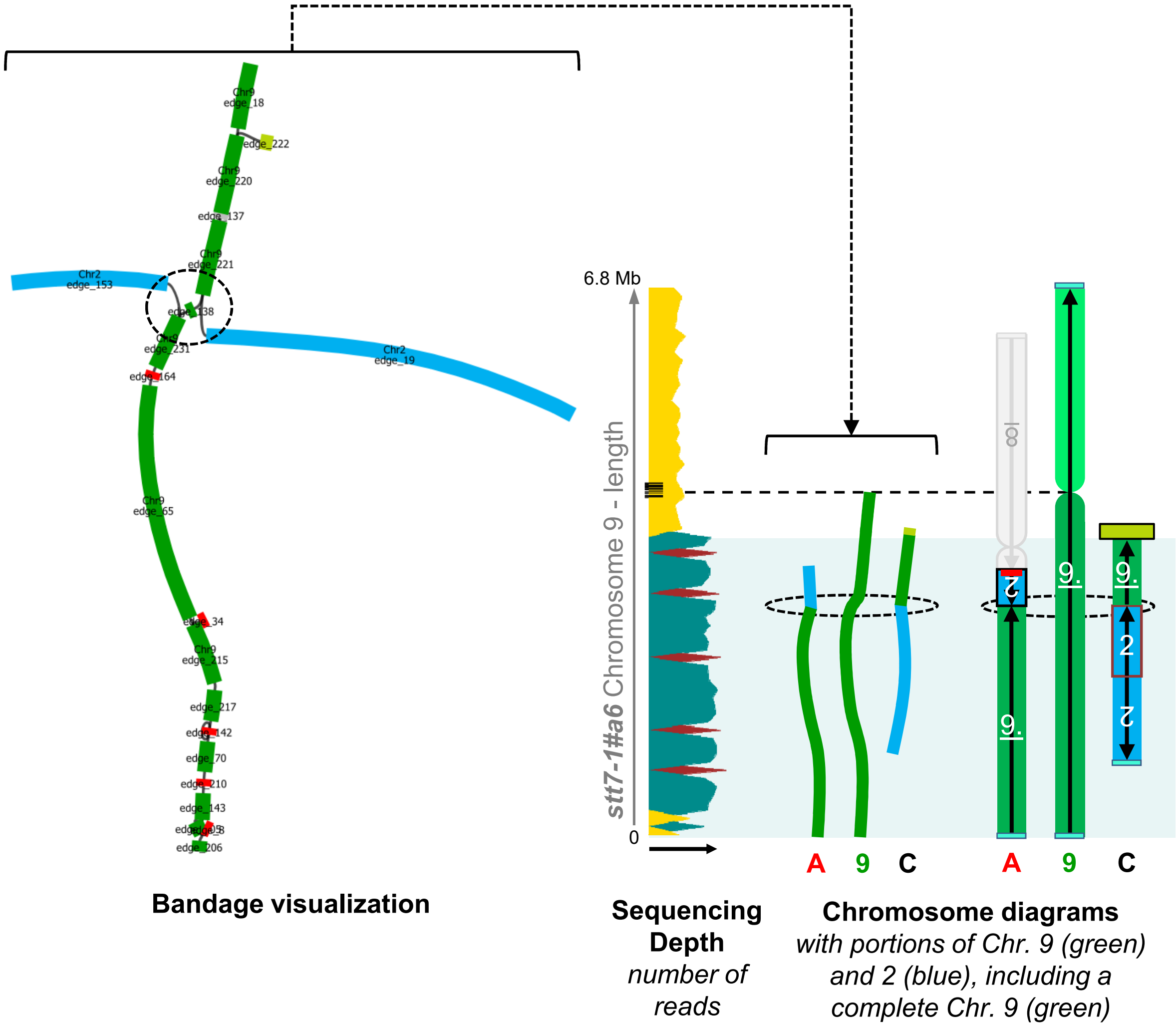

Figure S2. Chromosome 9 structure and node visualization with Bandage: Insights from *stt7-1 #a6*

On the left side, an example of visualization using Bandage is shown. This tool helps identify nodes where multiple sequences enter or exit of assembly. For instance, on Chr. 09 of *stt7-1 #a6*, a node is observed where two different segments of Chr. 02 are present.

On the right, the underlying gene structure of three chromosomes (A, 9 and C) in *stt7-1 #a6* is illustrated, based on the Bandage and D-GENIES (Supplemental Figure S1) visualizations.

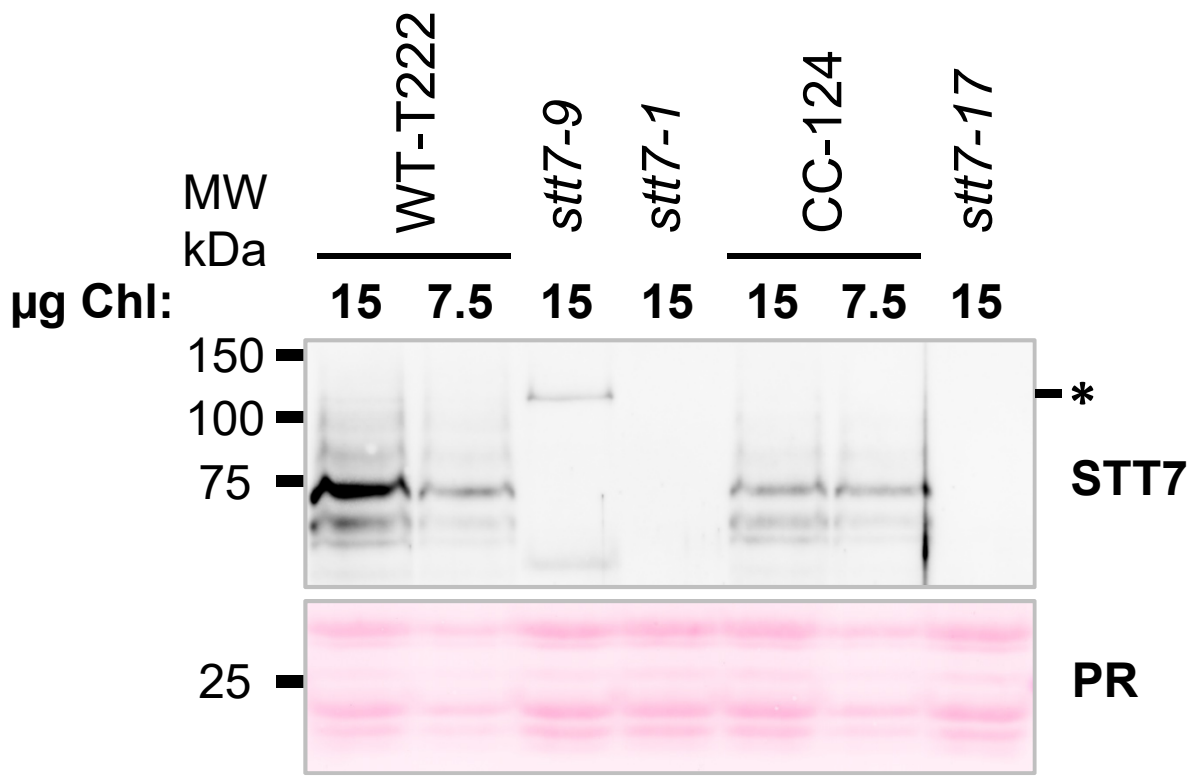

Figure S3: Immunoblot analysis of STT7 of the three *stt7* allelic mutants used in this study: thylakoids membranes were isolated (in state 1) and the proteins were separated by 12% SDS-PAGE gel (without urea), transferred to nitrocellulose membrane, and probed with antibodies against STT7. Asterisk (*) indicates the STT7-ARG7 fusion protein, detected only in *stt7-9*. Ponceau red staining (PR) is provided as loading control (correspond to LHCII region, ~25 kDa).

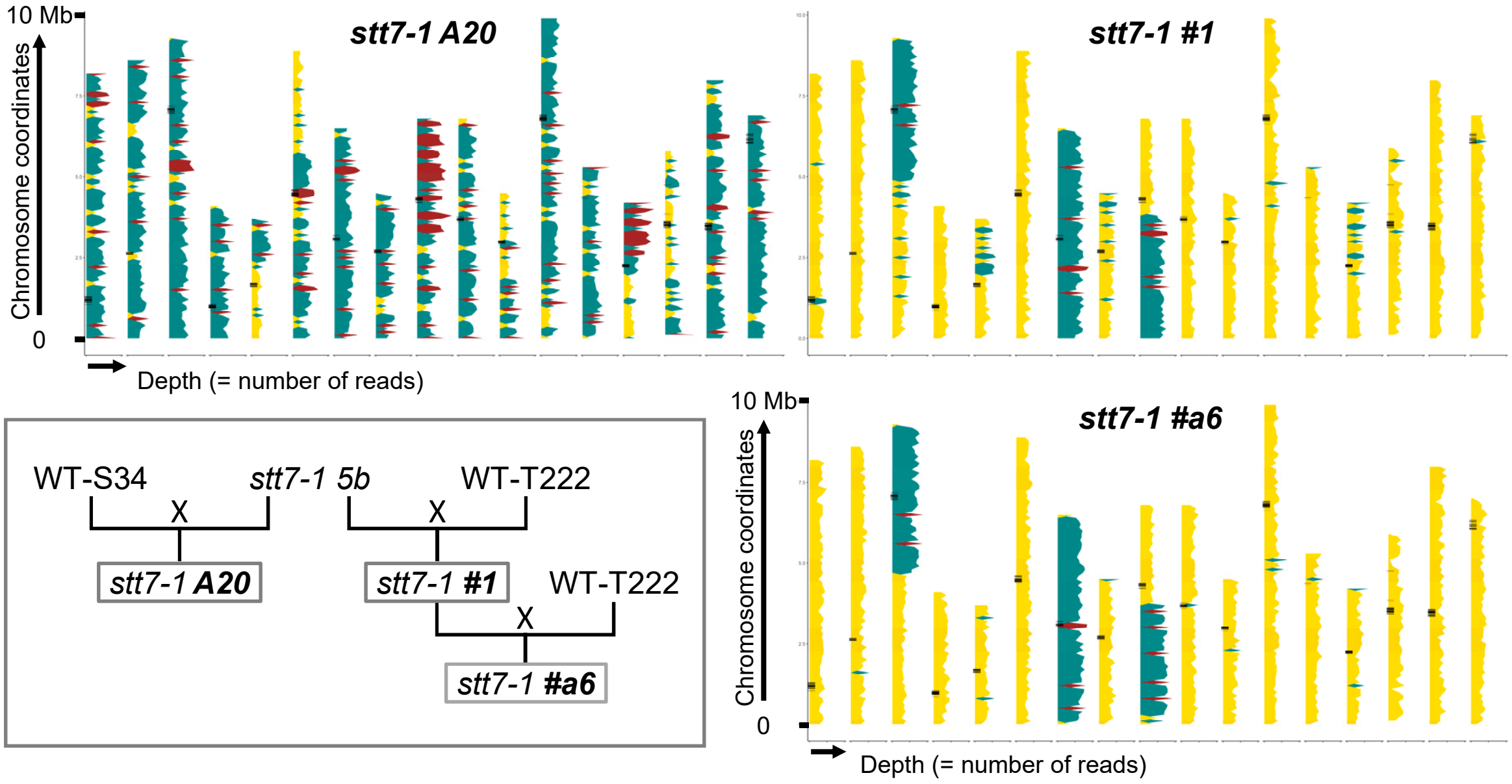

Figure S4. Long-read (Nanopore) whole-genome sequencing depth across each chromosome for three *stt7-1* strains. The strains were derived from backcrosses performed at IBPC (box at bottom left), using either WT-S34 (*mt*+) or WT-T222 (*mt*+) reference strain. Ploidy levels on the diagrams: haploid (1n) in yellow; diploid (2n) in dark cyan; triploid (3n) in red.

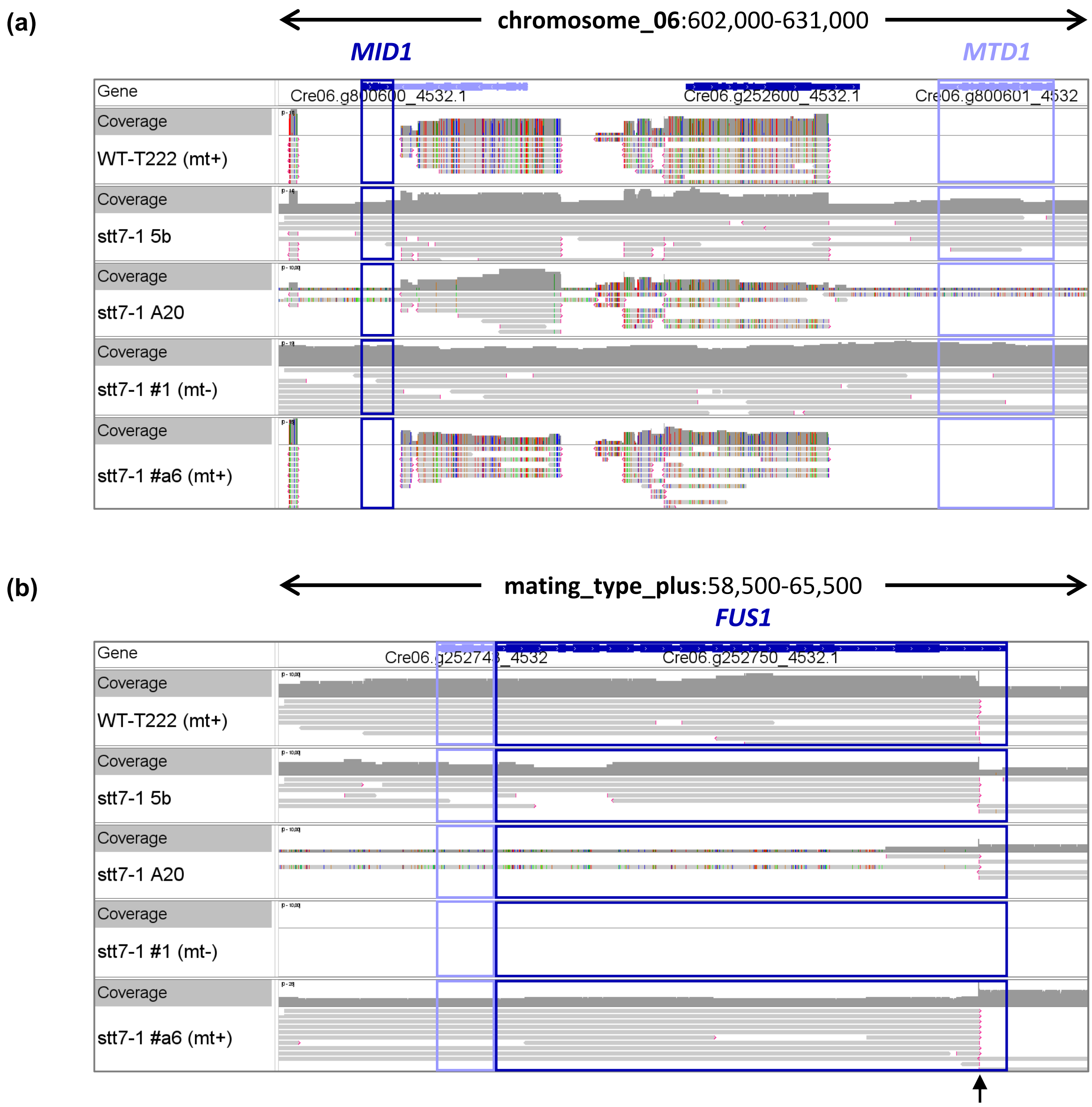

Figure S5. Sequencing coverage (dark grey) and read alignments (light grey) across two regions of the mating type (*MT*) locus, in different *stt7-1* and WT strains used in this study (visualized with IGV (Integrative Genomics Viewer)). Regions outlined in blue squares in **(a)** correspond to Cre06.g800600 (*MID1* - Minus dominance gene 1) and Cre06.g800601 (*MTD1* - MT locus, region d gene), which are Sex-limited to *mt-* strains, and in **(b)** to Cre06.g252743 and Cre06.g252750 (*FUS1*), *mt+* specific genes (exist only in the *mt+* locus). Colors on reads indicate variants relative to the reference genome (blue = C, green = A, gold = G, red = T). Black arrow indicates insertion site of *Gypsy 8* transposon in 3'UTR of *FUS1*.

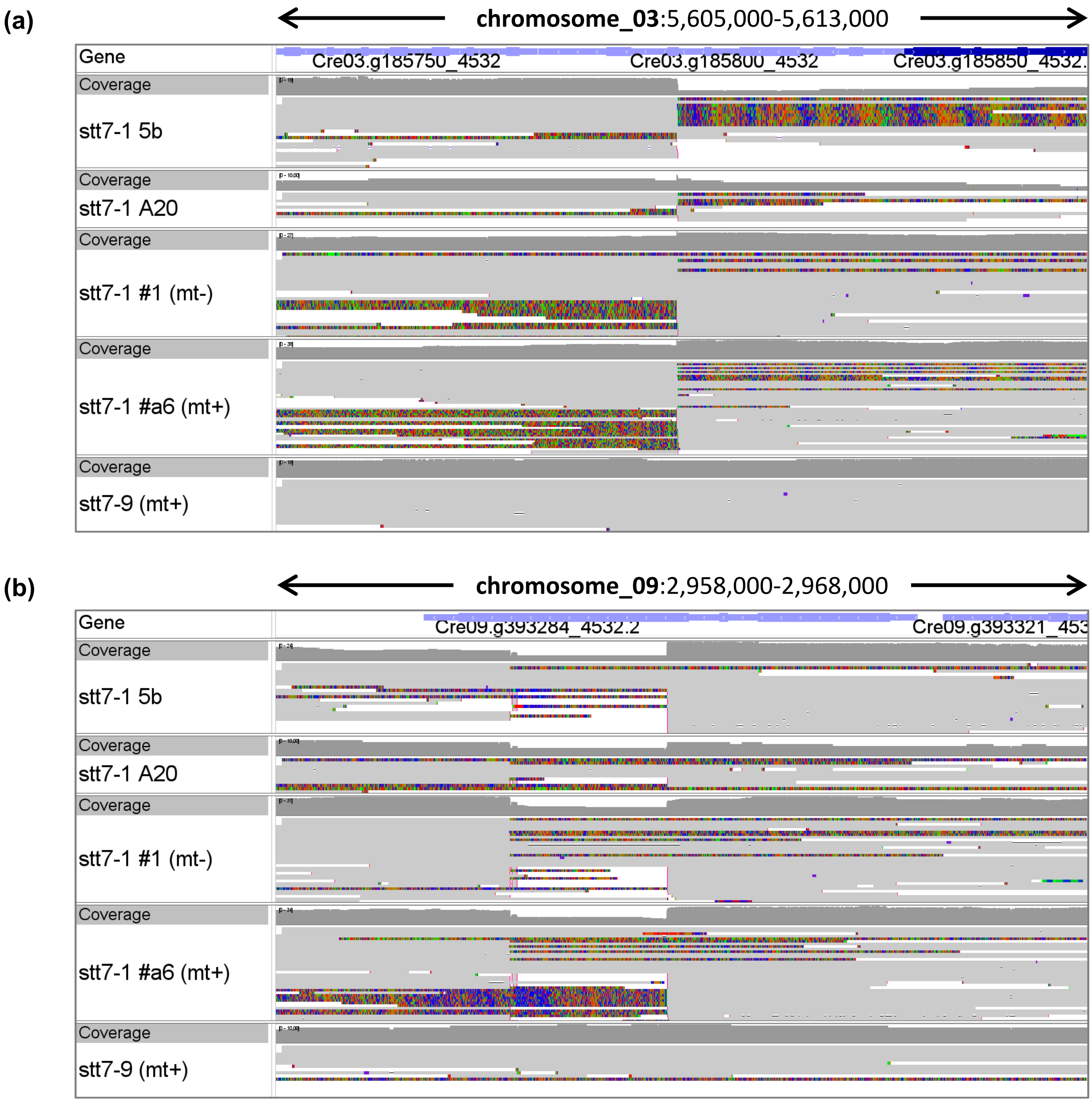

Figure S6. Sequencing coverage (dark gray) and read alignments (light gray) of two chromosomes regions in *stt7-1* and *stt7-9* strains (visualized with IGV (Integrative Genomics Viewer)). When the reads align perfectly with the reference sequence, they appear in light gray. Variations are shown as colored bases, representing differences from the reference genome (blue = C, green = A, gold = G, red = T). The presence of both types of reads for a given strain - completely gray reads and gray reads with a break or colored bases, starting at the same position (e.g., at the *Cre03.g185800* gene **(a)** or *Cre09.g393284* gene **(b)**)- reveals structural differences, confirming the co-existence of two haplotypes.

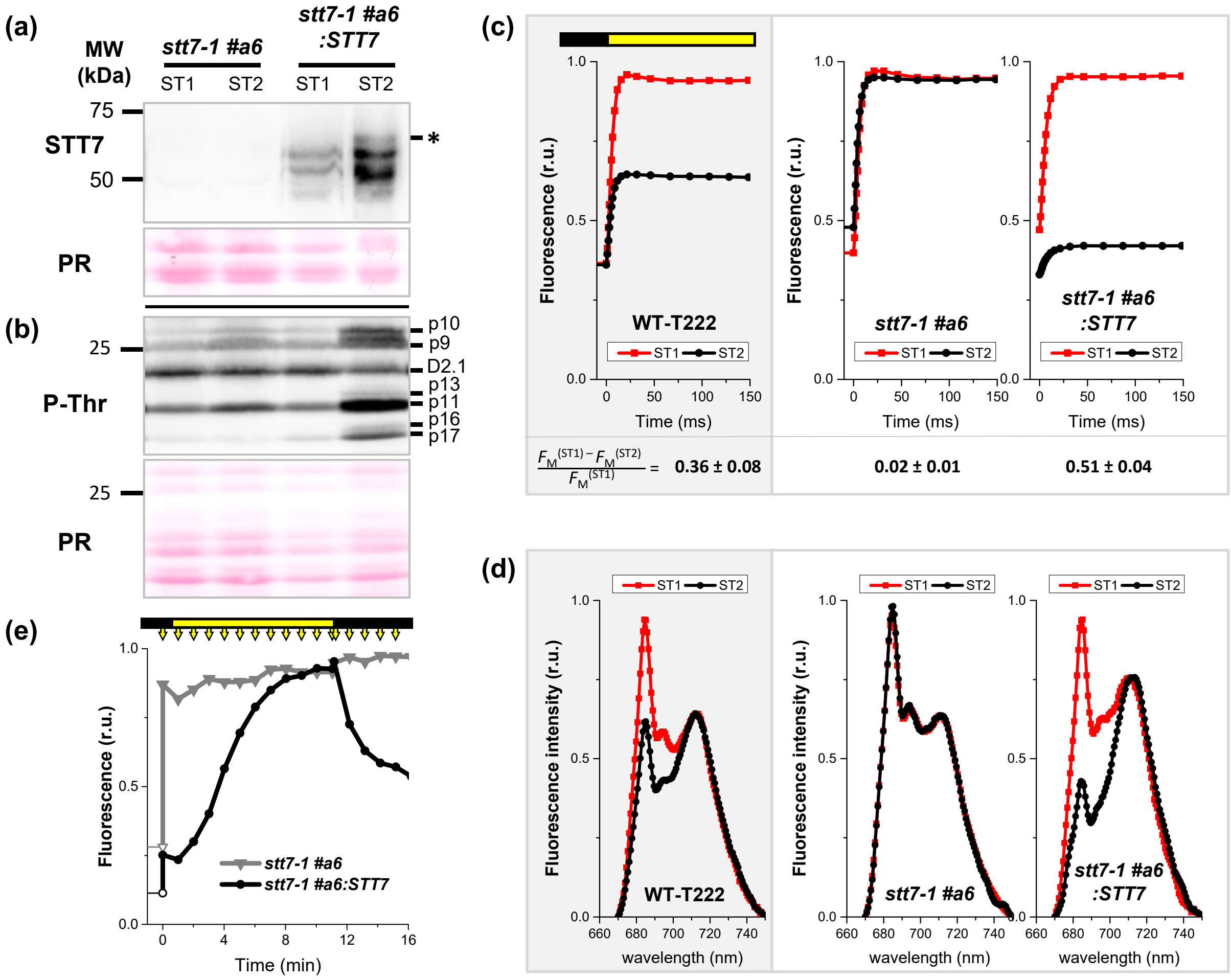

Figure S7. State Transitions characteristics of *stt7-1 #a6* clone and its complemented strain. (A-B) Immunoblot analysis of STT7 protein and phosphorylated polypeptides. Thylakoids membranes were isolated in state 1 or state 2 and the proteins were separated on a 8 M urea SDS-PAGE gel (12% -A- and 12% to 18% -B-), transferred to nitrocellulose membrane, and probed with antibodies against (A) STT7 and (B) phosphothreonine.

State transitions measurements by (C) emission fluorescence at room temperature in presence of DCMU, and **(D) low-temperature fluorescence emission spectra** (normalized to the PSI emission peak).

(E) Maximum fluorescence kinetic measurements of cell colonies from two paromomycin-resistant transformants, dark-adapted and maintained in a CO_2-enriched environment. The black curve represents a *STT7*-complemented transformant, showing a transition from state 2 to state 1 upon the dark-to-light shift, followed by a state 1-to-state 2 transition after the light is switched off. The first point (open) corresponds to F_0, and the second to F_M, both measured in darkness. The remaining points represent F_M values recorded under actinic light (160 µmol photons.m^{-2}.s^{-1}), following 10 successive saturating pulses at 1-minute intervals, with 5 additional measurements after the light is switched off. The gray trace corresponds to a measurement of the unrescued *stt7* transformant mutant grown on the same Petri dish under identical conditions.

On blot (A): asterisk (*) correspond to STT7 phosphorylated form; on blot (B): polypeptide bands p11/p13 correspond to LHCII type I (LHCBM3/4/6/8/9); p17 to LHCII type III (LHCBM2/7); p16 to LHCII type IV (LHCBM1); p9 to LHCB4; p10 to LHCB5; D2.1 to D2 (psbD) phosphorylated form. Ponceau Red staining (PR) are provided as loading control; in panel A, the LHCII region (~25 kDa) is displayed.

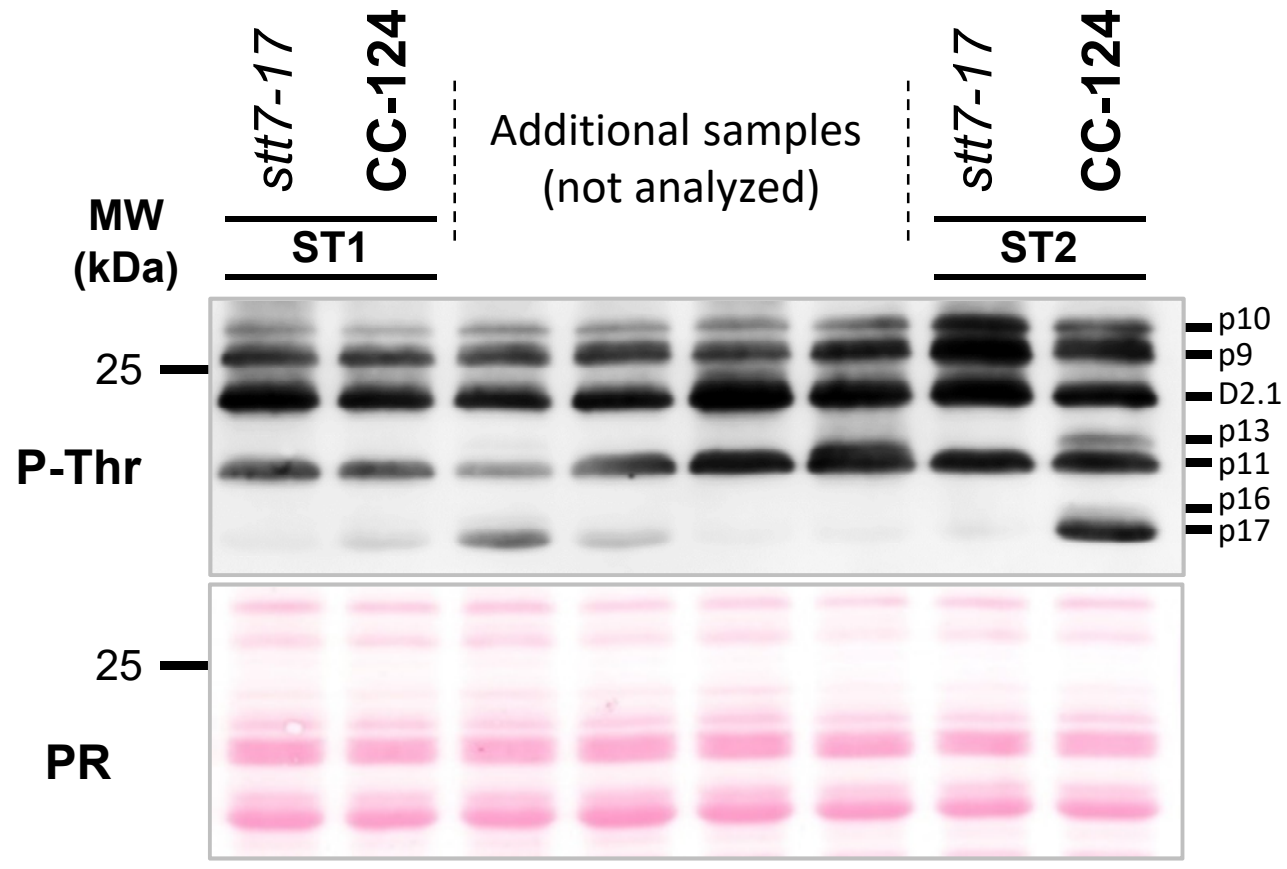

Figure S8. Immunoblot analysis of phosphorylated polypeptides. Thylakoids membranes were isolated in state 1 or state 2 and the proteins were separated on a 8 M urea SDS-PAGE gel (12% to 18%), transferred to nitrocellulose membrane, and probed with antibodies against phosphothreonine. Additional samples present on the membrane were not part of the present study.

p11/p13 correspond to LHCII type I (LHCBM3/4/6/8/9); p17 to LHCII type III (LHCBM2/7); p16 to LHCII type IV (LHCBM1); p9 to LHCB4; p10 to LHCB5; D2.1 to D2 (psbD) phosphorylated form. Ponceau red protein staining (PR) is provided as loading control.

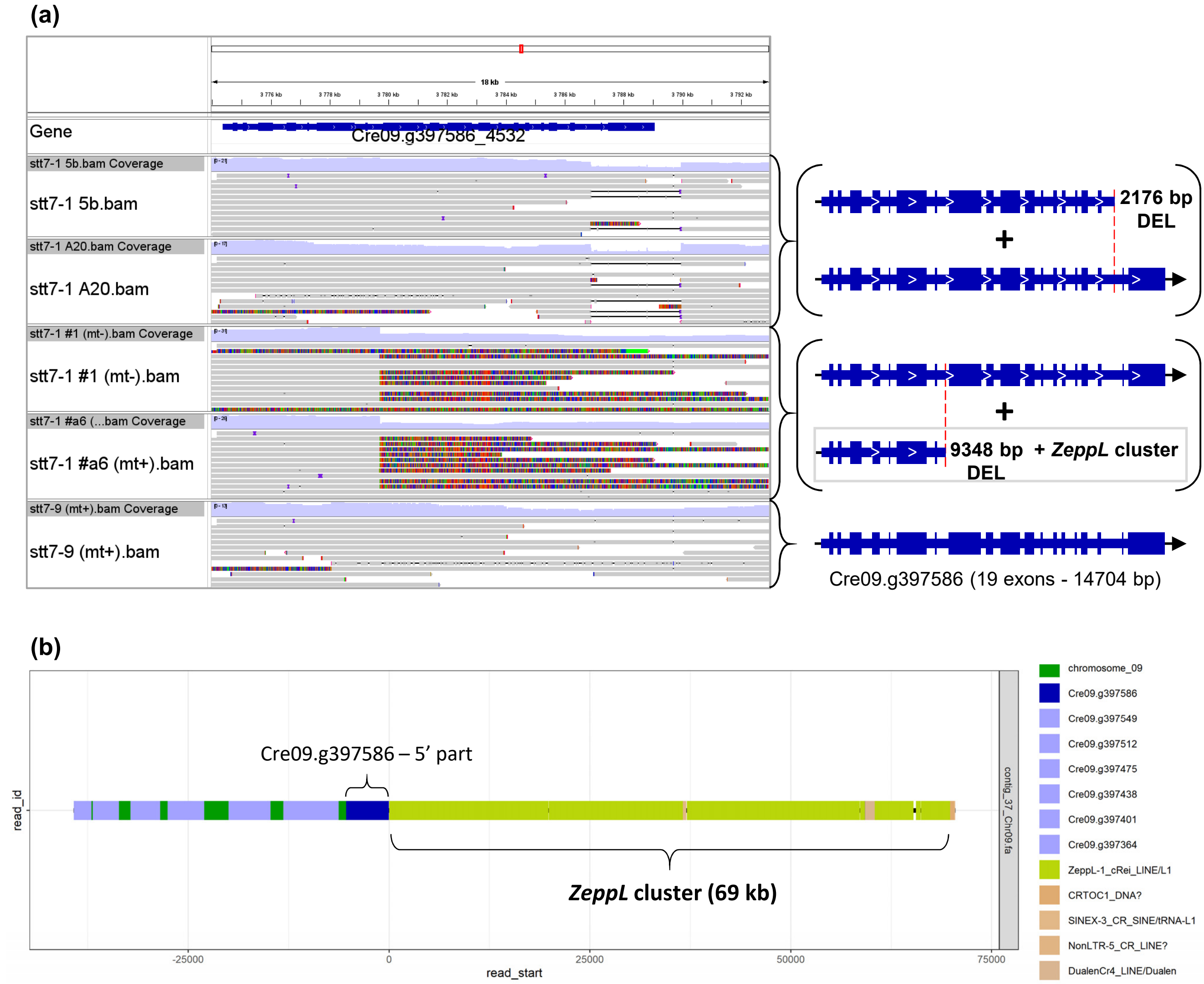

Figure S9. Cre09.g397586 gene structures in *stt7-1* and *stt7-9* strains.
(a) IGV (Integrative Genomics Viewer) visualization of mapped sequence reads on Cre09.g397586 (left), and schematic representation of gene variant across *stt7* strains (right). **(b)** *stt7-1 #a6* contig assembled from all reads using Flye, containing the *ZeppL* element sequence and the Cre09.g397586 gene sequence, identified by BLAST.

Table S1. Chlamydomonas strains used in this study.

Strain Name	Alternate Name	Genotype	Parental strains or Recipient strain	Availability
WT-S34		Wild type, *cgl46*, *mt+*	NA	ChlamyStation[1]
WT-T222	CC-5101	Wild type, *cgl46*, *mt+*	WT-S24, *mt-* X WT-N, *mt+*	ChlamyStation[1], Chlamydomonas Resource Center[2]
CC-124		Wild type, *mt-*	NA	Chlamydomonas Resource Center[2]
CC-125		Wild type, *cgl46*, *mt+*	NA	Chlamydomonas Resource Center[2]
stt7-9	CC-5682	*stt7*, *cgl46, mt+*	NA	ChlamyStation[1], Chlamydomonas Resource Center[2]
stt7-9		*stt7*, *cgl46, mt-*	NA	ChlamyStation[1]
stt7-1 5b		*stt7*, CGL46/*cgl46, mt+/mt-*	NA	ChlamyStation[1]
stt7-1 A15		*stt7*, CGL46/*cgl46, mt+/mt-*	*stt7-1 5b* X WT-S34	ChlamyStation[1]
stt7-1 A20	CC-5681	*stt7*, CGL46/*cgl46, mt+/mt-*	*stt7-1 5b* X WT-S34	ChlamyStation[1]
stt7-1 #1		*stt7, mt-*	*stt7-1 5b* X WT-T222	ChlamyStation[1]
stt7-1 #a6		*stt7, mt+*	*stt7-1 #1* X WT-T222	ChlamyStation[1]
stt7-17	CC-6272	*stt7, cgl46, mt+*	<u>CC-125</u>	Chlamydomonas Resource Center[2]
stt7-17 #a3		*stt7, mt+*	*stt7-17* X CC-124	ChlamyStation[1]
CLiP2.022035		*Cre09.g387023, mt+*	<u>CC-5415, *mt+*</u>	Chlamydomonas Resource Center[2]
CLiP2.031783		*Cre09.g387023, mt+*	<u>CC-5415, *mt+*</u>	Chlamydomonas Resource Center[2]

NA, not applicable.
[1] ChlamyStation, IBPC, Paris, FR, http://chlamystation.free.fr/
[2] Chlamydomonas Resource Center, University of Minnesota, USA, https://www.chlamycollection.org/

Table S2. Primers used for the PCR experiments

Target gene	Primer name	Sequence 5'-3'	Amplicon size
MID	OLIM1<br>OLIM2	TGGCGTACCTTTCTGTAGGG<br>GCCACGAAGGCAGTTACATT	498 bp
FUS1	OLIP1<br>OLIP2	CCGCACATGAGACGTTACAG<br>GATTGCTCTGTCGTTGCAGA	381 bp
CGL46	F1_CK CGL46<br>RA_CGL46	CGACTTCGTGGAGGCCTTCATGC<br>TGCGCCTTGTCCACGTTGAT	622 bp
gypsy-7a<br>*(in CGL46)*	F1_CK CGL46<br>R2_GYPSY-7a	CGACTTCGTGGAGGCCTTCATGC<br>CGGTGACTCTACTTGGGCAACC	961 bp
STT7 (5')<br>*(exon1→ exon4)*	F_STT7-CK<br>R_STT7-CK	TGCCGCATTGAGCGGTCTACA<br>CGCTTGCCCAGGACAATGTCAT	1166 bp
STT7<br>*(full-length gene)*	2F1_STT7<br>2R6_STT7	AATGTACGATGGTGTCCAGC<br>TTAGGTCACCTCGTGCAACT	8850 bp
